# Down-Regulation of GABA_A_ Receptor *via* Promiscuity with the Vasoactive Peptide Urotensin II Receptor. Potential Involvement in Astrocyte Plasticity

**DOI:** 10.1371/journal.pone.0036319

**Published:** 2012-05-01

**Authors:** Laurence Desrues, Thomas Lefebvre, Céline Lecointre, Marie-Thérèse Schouft, Jérôme Leprince, Vincent Compère, Fabrice Morin, François Proust, Pierrick Gandolfo, Marie-Christine Tonon, Hélène Castel

**Affiliations:** 1 Inserm U982, Laboratory of Neuronal and Neuroendocrine Communication and Differentiation, Astrocyte and Vascular Niche, University of Rouen, Mont-Saint-Aignan, France; 2 Institute of Research and Biomedical Innovation (IRIB), Normandy University PRES, University of Rouen, Mont-Saint-Aignan, France; 3 Department of Anesthesiology and Critical Care, Rouen University Hospital, Rouen, France; 4 Department of Neurosurgery, Rouen University Hospital, Rouen, France; Biological Research Centre of the Hungarian Academy of Sciences, Hungary

## Abstract

GABA_A_ receptor (GABA_A_R) expression level is inversely correlated with the proliferation rate of astrocytes after stroke or during malignancy of astrocytoma, leading to the hypothesis that GABA_A_R expression/activation may work as a cell proliferation repressor. A number of vasoactive peptides exhibit the potential to modulate astrocyte proliferation, and the question whether these mechanisms may imply alteration in GABA_A_R-mediated functions and/or plasma membrane densities is open. The peptide urotensin II (UII) activates a G protein-coupled receptor named UT, and mediates potent vasoconstriction or vasodilation in mammalian vasculature. We have previously demonstrated that UII activates a PLC/PIPs/Ca^2+^ transduction pathway, *via* both G_q_ and G_i/o_ proteins and stimulates astrocyte proliferation in culture. It was also shown that UT/G_q_/IP_3_ coupling is regulated by the GABA_A_R in rat cultured astrocytes. Here we report that UT and GABA_A_R are co-expressed in cerebellar glial cells from rat brain slices, in human native astrocytes and in glioma cell line, and that UII inhibited the GABAergic activity in rat cultured astrocytes. In CHO cell line co-expressing human UT and combinations of GABA_A_R subunits, UII markedly depressed the GABA current (β_3_γ_2_>α_2_β_3_γ_2_>α_2_β_1_γ_2_). This effect, characterized by a fast short-term inhibition followed by drastic and irreversible run-down, is not relayed by G proteins. The run-down partially involves Ca^2+^ and phosphorylation processes, requires dynamin, and results from GABA_A_R internalization. Thus, activation of the vasoactive G protein-coupled receptor UT triggers functional inhibition and endocytosis of GABA_A_R in CHO and human astrocytes, *via* its receptor C-terminus. This UII-induced disappearance of the repressor activity of GABA_A_R, may play a key role in the initiation of astrocyte proliferation.

## Introduction

Integrated brain function and dysfunction arise from the complex interactions between a network of multiple cell types including neurons, c and the microvascular endothelial cells comprising the cerebral vasculature [1], [2], [3]. This micro-environment is a dynamic structure referred as neurovascular unit where polarized astrocytes have a pivotal role [4], rapidly transducing synaptic information [2], [3], [4], [5]. In pathological conditions including stroke, the astroglial reactivity is characterized by proliferation, hypertrophy, process extension, increased synthesis of intermediate filaments, as well as expression of bioactive molecules and their receptors [6], [7], [8].

GABA_A_ receptors (GABA_A_R) are believed to be pentameric heterooligomers mainly constructed from homologous subunit types α_1–6_, β_1–3_, γ_1–3_, δ and ε [9], [10], [11]. The GABA_A_R is expressed in neurons but also in glial cells in culture [12], brain slices [13], acutely isolated hippocampal slices [13], membrane fractions of the mature rodent brain [14] and also *in vivo* in healthy brain [15]. In pathological conditions, a significant decrease of benzodiazepine sites associated to the GABA_A_R has been demonstrated in patients with ischemic cerebrovascular [15], [16], [17], Parkinson [18] and Alzheimer [19], [20] diseases. It was also observed a reduced chloride conductance [21], a decrease in receptor mediated inhibitory post-synaptic potentials [22] and a marked down-regulation of the GABA_A_R expression at the cell surface along with a fast time course [15], [23], [24]. In reactive and malignant astrocytes, mRNA levels of GABA_A_R have been shown to remain constant before diminution of functional GABA_A_R [15], [25]. Thus, the disappearance of GABA_A_R expression is correlated with higher glial proliferation rate after stroke or during malignancy of astrocytoma [15], [25], [26], leading to the hypothesis that GABA_A_R expression/activation works as a repressor of cell proliferation. Investigations on alterations in GABA_A_R-mediated functions, receptor densities or modulation in astrocytes remain unchallenged. It has been demonstrated that simultaneous activation of different postsynaptic receptors induces cross-modulation of their activation properties and receptor membrane insertion/deletion. Thus, as many neurotransmitters and vasoactive peptides are released by endothelium and astrocytes, and their receptors are expressed by astrocytes, there is a potential for complex signaling within the neurovascular unit, involving receptor cross-talks.

Urotensin II (UII) and its paralog urotensin II-related peptide URP, are highly efficient vasoactive peptides, which share a fully conserved C-terminal cyclic CFWKYC core corresponding to the molecular pharmacophore [26], [27], [28]. The biological actions of UII and URP are mediated through activation of a G protein-coupled receptor named UT. It is now clearly established that activation of native UII receptors or UT-transfected cell lines is associated with an increase in polyphosphoinositide (PIPs) turn-over promoting a cytosolic calcium concentration ([Ca^2+^]c) rise [29], [30], [31]. UII and UT are expressed in the mammalian cardiovascular system namely in the myocardium, vascular smooth muscle cells and endothelial cells [32], [33], [34], [35], affecting cell proliferation [35], [36] or neoangiogenesis [37], stimulating collagen synthesis and cardiac hypertrophy [34].

In the brain, UII mRNA is particularly abundant in motoneurons of the medulla oblongata and spinal cord [38], [39] while UT mRNA is widely expressed in various regions of the central nervous system including the olfactory system, hippocampus, amygdala, hypothalamus, or cerebellum [27]. However, immunohistochemical studies revealed that UT is expressed in astroglial processes *in vivo* [40] and in cultured rat cortical astrocytes [41]. In this cell type, we have previously demonstrated that UII activates a PLC/PIPs/Ca^2+^ transduction pathway, *via* both G_q_ and G_i/o_ proteins and stimulates cell proliferation [41], [42]. Moreover, a functional interaction between GABA_A_R and UT suggested a cross-talk between these two receptors, involved in astrocyte activity [43]. In this study we demonstrate that activation of UT receptor induces a long-term inhibition of GABA_A_R-mediating chloride currents, a process potentially relevant for astrocyte proliferation.

## Results

### GABA_A_R and UT functional coupling in rat cerebellar astrocytes

**Figure 1:**
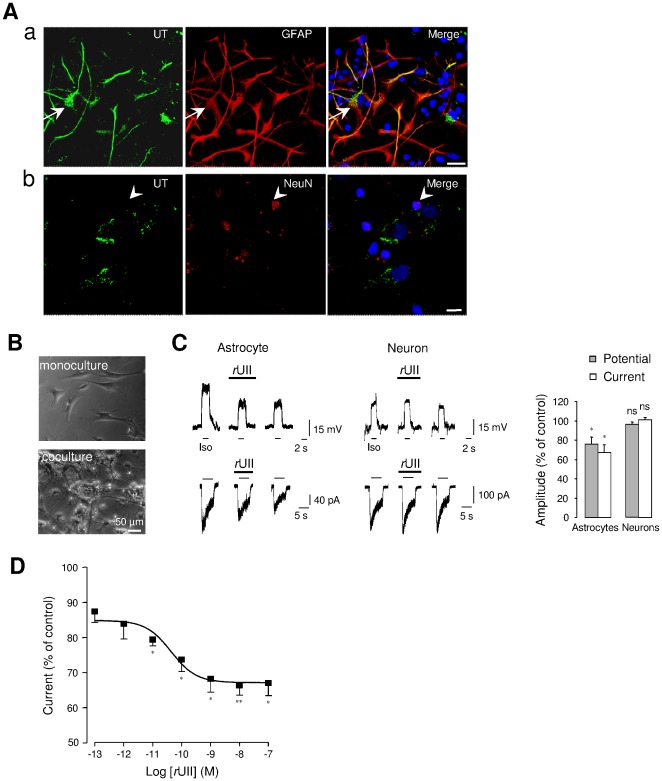
UII-induced depression of GABA_A_R in UT-expressing cerebellar astrocytes. (Aa, Ab) Double immunofluorescence labeling of UT (green) and the specific astrocyte marker GFAP (red, Aa), or the mature neuron marker NeuN (red, Ab) in astrocyte-neuron co-culture from P7 rat cerebellum. Astrocytes, recognized by strong GFAP staining show UT immunoreactivity (arrows), whereas few weaker UT-stained cells express NeuN (arrowheads), and were likely attributed to mature granule cells (arrowheads, Ab). Nuclei (blue) were counterstained with DAPI. Scale bars, 50 µm. (B) Phase contrast photomicrograph of astrocytes in mono-culture, or astrocytes and neurons in co-culture at 3 days *in vitro*. (C) Membrane depolarizations and currents evoked by the GABA_A_R agonist isoguvacine (Iso, 10^−4^ M, 2 s for membrane potential and 5 s for chloride current) in astrocytes and cerebellar granule neurons before, during *r*UII (10^−7^ M, 40 s) application and after 2-min washout. Right, normalized amplitudes deduced by the mean Iso-evoked depolarization or current obtained before *r*UII application. (D) Concentration-response relationship of Iso-evoked currents from astrocytes yielding an EC_50_ value of 43.6±23.7 10^−12^ M. Data are mean ± SEM of 4 to 6 cells. *, *P*<0.05; ** *P*<0.01 compared with the corresponding control Iso-evoked current.

We and others have previously demonstrated that astrocytes express *in vitro* and *in vivo* UT mRNA and/or protein [38], [40], [41]. As shown in Figure 1Aa and 1b, UT is extensively expressed in astroglial processes, but also in the sparse mature granule cells present in co-cultures. In order to investigate a potential cross-talk between GABA_A_R and UT in both astrocytes and neurons, patch-clamp recording of the GABA_A_R agonist isoguvacine (Iso, 10^−4^ M) responses was carried out on membrane potentials and currents of astrocytes in mono-culture, and astrocytes or neurons in co-culture (Figure 1B and 1C). We established that flat and proliferating astrocytes in mono-culture (*n* = 31) (Figure 1B) were rarely responding (only 12% of cells are responding to Iso). In contrast, astrocytes cultured with cerebellar granule neurons present a slowly proliferating stellate astrocytic morphology (*n* = 78) (Figure 1B) and are more responding to the GABA_A_R agonist (48% of tested cells). In co-cultured astrocytes, local perfusion of rat UII (*r*UII, 10^−7^ M, 40 s) provoked a marked inhibition of the amplitude of the depolarization and chloride current (voltage clamp; −60 mV) evoked by the GABA_A_R agonist isoguvacine by 24.2±7.5% and 33±8% (*n* = 5), respectively (Figure 1C). In contrast, *r*UII did not affect the Iso-evoked depolarization and current in cerebellar granule neurons (Figure 1C). In astrocytes, *r*UII induced a dose-dependent inhibition of the current with an EC_50_ value of 43.6±23.7% pM (Figure 1D). It can be noticed that in some cells, UII tested at 10^−8^ M, activated a small inward current. These data indicate that in astrocytes, unlike in neurons, *r*UII efficiently and markedly down regulated the GABAergic activity when UT and GABA_A_R are co-expressed.

**Figure 2:**
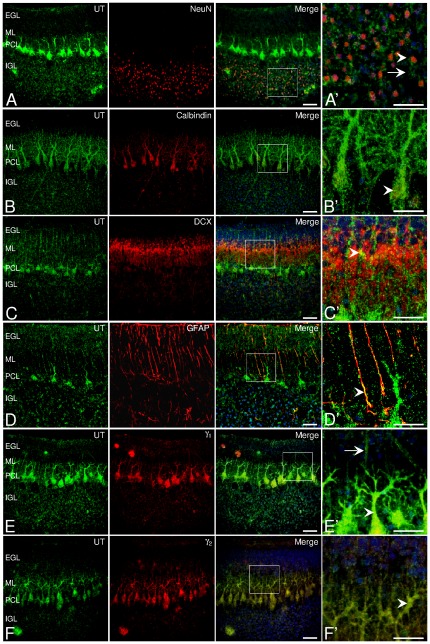
Co-localization of UT with γ subunits in neuron and glial components in rat cerebellum. (A, A′) Double-fluorescence staining for UT (green) and NeuN (red) showing the presence of UT in both mature (arrowhead, merge, A′) and unidentified cells (arrows, merge, A′) in the IGL. (B) Co-staining of UT and the marker of Purkinje cells, calbindin (red), in Purkinje cell soma and dendrites (arrowhead, B′). (C) Staining for UT and the marker of migrating neuroblasts doublecortin DCX (red) depicting a diffuse labeling in the ML. (C′) UT immunopositive fibers contiguous to DCX-expressing migrating granule cells (merge, yellow, arrowhead). (D, D′) Staining for UT and GFAP (red) in glial fibers (merge, yellow, arrowhead) of the ML. (E, F) Distribution of UT and the γ_1_ (E) and γ_2_ (F) GABA_A_R subunits (red), in Purkinje cells (merge, arrowhead) and few extents of glia (merge, arrow) in the ML and IGL. Nuclei (blue) were counterstained with DAPI. Scale bars, 50 µm (A–F); 20 µm (A′–F′). EGL, external granule cell layer; IGL, internal granule cell layer; ML, molecular layer; PCL, Purkinje cell layer. (A′–F′) images of digitally zoomed regions corresponding to the white boxes in A–F.

In postnatal day 7 (P7) cerebellar slices, we investigated the topographic cellular and subcellular distribution of UT in the different layers. UT protein immunoreactivity was specifically distributed in particular zones of the cerebellar cortex, in the Purkinje cell layer (PCL), on fibers irradiating from the thin molecular cell layer (ML) toward the external granule cell layer (EGL), and on isolated cells residing in the internal granule cell layer (IGL) (Figure 2). In particular, UT receptor staining was present on NeuN-positive mature granule cell bodies (Figure 2A and 2A′) and also in Purkinje neuronal cell bodies and ramifications, as revealed by the strong immunofluorescence of UT in calbindin-positive Purkinje cell soma and dendrites (Figure 2B and 2B′). Doublecortin (DCX) is a distinctive marker of granule cells during the period of radial descent along the Bergmann glia into the cerebellar layers [44]. The DCX immunoreactivity appeared as a diffuse labeling in the IGL and densely in the ML, contiguously associated to UT-positive components but not co-localized (Figure 2C and 2C′). Moreover, UT was strongly co-localized with GFAP expressed by astrocytes of the white matter (*not shown*), and on glial cells (Figure 2D) including Bergmann cells [45]. At higher magnification, the double immunofluorescence analysis revealed that Bergmann cell bodies and fibers were surrounded by yellow co-localizing signals of UT varicosities and of GFAP-positive filaments (Figure 2D′). In addition, immunohistochemical analysis showed labeling of the γ_1_ subunit in Purkinje cells, and faint staining in fibers of the ML and in granule cell bodies of the IGL, co-localizing with UT (Figure 2E and 2E′). The γ_2_ subunit immunoreactivity was also mainly detected in Purkinje cells and fibers of the EGL (Figure 2F and 2F′). Thus, it appears that UT and GABA_A_R subunits are colocalized in cerebellar Purkinje and glial cell fibers *in situ*.

### UT mediates inhibition of γ-composed GABA_A_R complexes

**Figure 3:**
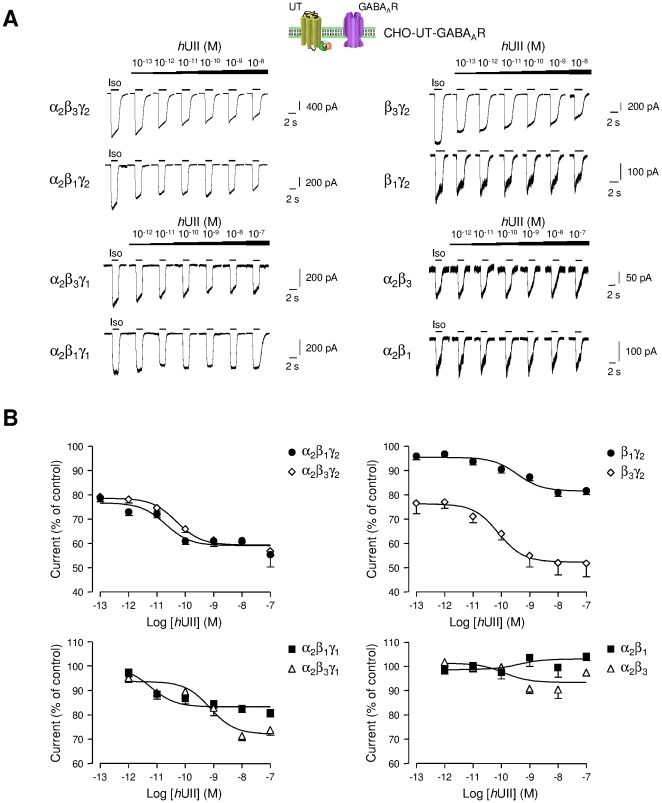
Effect of *h*UII on different GABA_A_R subunit combinations. (A) Typical Iso-evoked currents at the holding potential of −60 mV, in the whole-cell configuration, on CHO stably expressing human UT (CHO-UT) and transiently transfected with cDNAs encoding α_2_β_3_γ_2_, α_2_β_1_γ_2_, α_2_β_3_γ_1_, α_2_β_1_γ_1_, β_3_γ_2_, β_1_γ_2_, α_2_β_3_ or α_2_β_1_ subunits of the GABA_A_R. Iso (10^−4^ M) was repeatedly applied for 2 s at 2 min intervals and increasing concentrations of *h*UII (10^−13^ to 10^−7^ M) were bath perfused in the vicinity of cells. (B) Corresponding concentration-response curves for *h*UII on α_2_β_1_γ_2_ and α_2_β_3_γ_2_, α_2_β_1_γ_1_ and α_2_β_3_γ_1_, β_1_γ_2_ and β_3_γ_2_, α_2_β_1_ and α_2_β_3_ receptor subunits. Data are normalized to the control Iso response immediately prior to lower *h*UII concentration application. Data are mean ± SEM of 3 to 23 cells.

The molecular basis for the observed directional decrease in GABA_A_R function has been investigated in CHO co-expressing human UT and αβ and/or γ GABA_A_R subunits. GABA_A_R can be made from several different subunit families (α_1_–α_6_, β_1_–β_3_, γ_1_–γ_3_, ρ_1–3_, δ, ε, π, and θ), which come together in various combinations to form the pentameric receptor [46]. Most receptors are thought to contain α, β and the third subunit type varying, being often the γ_2_. Expression of unitary subunits has produced conflicting results, but some subunits expressed alone [47], [48] or as binary combinations, for instance α_1_γ_2_ or β_2_γ_2_, appear to be able to produce GABA-gated ions channels [49], [50], [51]. In order to establish a direct functional link between GABA_A_R and UT, the effect of graded concentrations of *h*UII was studied on the current evoked by Iso on CHO-UT, expressing different subunit combinations of GABA_A_R (Figure 3A). It is observed that *h*UII induced inhibition of GABA_A_R complexes which systematically contained γ_1_ or γ_2_ subunit (Figure 3A and 3B), and the normalized data fit yielded various EC_50_ values and efficacies summarized in supplementary information (Table S1). In particular, *h*UII was less effective on the α_2_β_3_γ_1_ and α_2_β_1_γ_1_ and totally ineffective on the α_2_β_3_ and α_2_β_1_ complexes (Figure 3A and 3B, Table S1).

**Figure 4:**
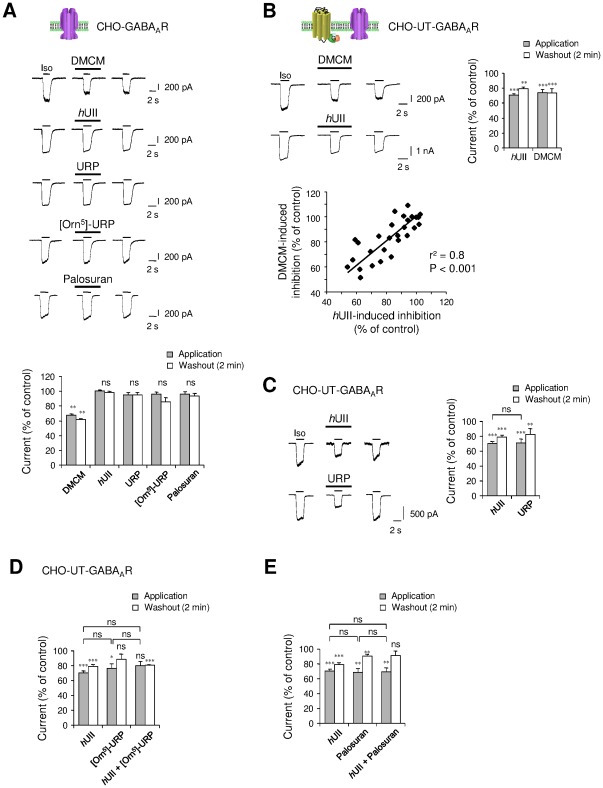
Pharmacological characterization of the UT-mediated inhibition of the GABA_A_R currents. (A) Whole-cell current response to Iso (10^−4^ M, 2 s) recorded in the absence or presence of the benzodiazepine site inverse agonist DMCM (10^−5^ M), *h*UII and URP (10^−8^ M, each), or UT antagonists [Orn^5^]-URP and palosuran (10^−6^ M, each) in CHO expressing α_2_, β_3_ and γ_2_ subunits. Below, summary of the various experimental conditions (*n* = 3–18). (B) Comparison of the inhibitory effect of *h*UII and DMCM on CHO-UT-GABA_A_R, as summarized in bar graphs (*n* = 25). Bottom row, plot of the positive correlation (r^2^ = 0.8) of *h*UII-induced inhibition as function of the DMCM-evoked current decrease (*n* = 28). (C) Comparison of the inhibitory effect of *h*UII and URP on CHO-UT-GABA_A_R as summarized in bar graphs (*n* = 12–54). (D, E) Effect of [Orn^5^]-URP and palosuran in the absence or presence of *h*UII *versus* the effect of *h*UII alone. Right, summary of the various experimental conditions (*n* = 7–54). Data are mean ± SEM from 3 to 54 cells. ns, non significant, *, *P*<0.05; ** *P*<0.01; *** *P*<0.001 compared with the corresponding control Iso-evoked current.

As a control, the effect of the GABA_A_R allosteric inverse modulator DMCM, *h*UII and other urotensinergic modulators, were tested on the α_2_β_3_γ_2_ GABA_A_R function in the absence of UT. Our data demonstrated that, as expected, DMCM induced inhibition of the current in most tested cells, and *h*UII and its paralog URP failed to affect the amplitude of the current (Figure 4A). The rapid development in recent years of several UT antagonists has led to the synthesis of [Orn^5^]-URP which has been previously characterized in our laboratory [52], [53] and palosuran, with one such high affinity toward human UT [54], [55]. Thus, the specific rat UT antagonist [Orn^5^]-URP (10^−6^ M), and the primate UT specific antagonist palosuran (10^−6^ M), did not modulate the Iso-evoked current.

To assess the role of γ subunit into αβ binary complexes in the UII-induced GABA_A_R regulation, we tested the effect of DMCM, as this allosteric modulator exhibits negative effect on GABA current in cells expressing γ_2_ subunit [56], [57] or null effect when γ is not present [56]. We analyzed the effect of *h*UII (10^−8^ M) concomitantly to that of DMCM (10^−6^ M) on CHO-UT co-expressing α_2_β_3_γ_2_ GABA_A_R. The amplitude of the *h*UII-induced inhibition was plotted as a function of the DMCM-induced inhibition of chloride currents recorded from twenty five CHO-UT-GABA_A_R expressing cells. A linear regression analysis confirmed the significance of a correlation as represented on the scatter plot (r^2^ = 0.8, *P*<0.001) (Figure 4B), suggesting that γ_2_ regulates the degree of UT-mediating inhibition of the GABAergic activity.

The effects of the different urotensinergic ligands were then tested on CHO-UT-GABA_A_R composed of the γ_2_ subunit. We found that *h*UII and URP (10^−8^ M, each) induced a marked current inhibition by 29.24±1.90% (*n* = 54) and 22.9±3.5% (*n* = 12), respectively, that persisted during washout for *h*UII, but slightly recovered during washout for URP (Figure 4C). In order to examine whether UT antagonists might counteract the UII-induced decrease of the GABAergic activity, [Orn^5^]-URP and palosuran were tested. [Orn^5^]-URP (10^−6^ M) significantly, but weakly inhibited the Iso-evoked current, blocked the effect of the acute application of *h*UII, but failed to counteract the prolonged effect of the peptide on the same cell (Figure 4D). Surprisingly, palosuran mimicked the *h*UII-induced inhibition of the Iso-evoked current, but abolished the irreversible action of *h*UII during washout (Figure 4E). This indicates that [Orn^5^]-URP or palosuran may keep their antagonist profile toward UT, but exhibit “agonistic activity" in regard to the GABA_A_R function.

**Figure 5:**
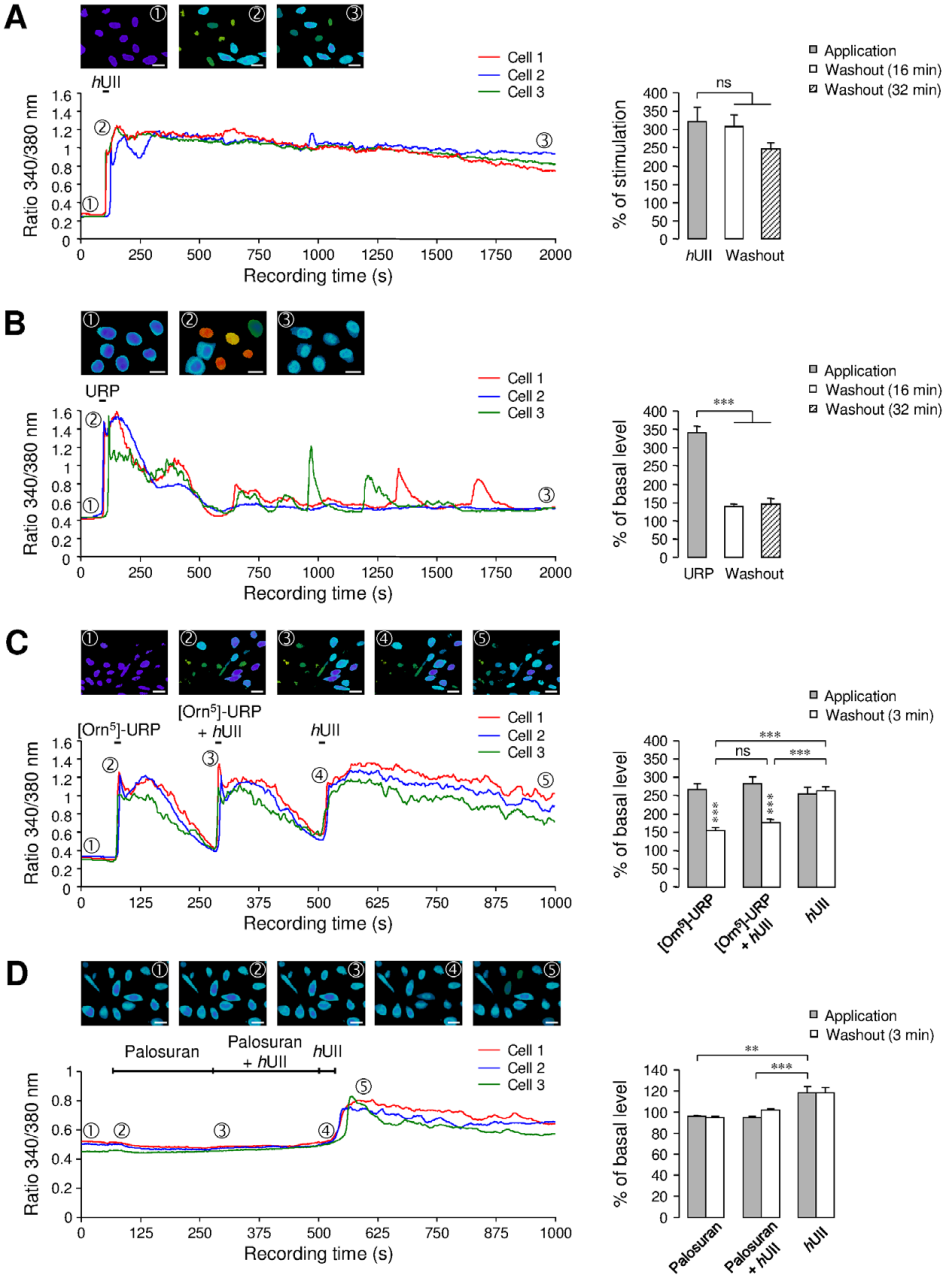
Role of specific UT ligands on cytosolic calcium in CHO-UT. (A, B) *h*UII (A) or URP (B) (10^−8^ M, each) provoked a robust increase of [Ca^2+^]_c_, which remained stable (A) or recovered to the basal line level (B) during washout. (C, D) Effect of the UT antagonists [Orn^5^]-URP (10^−6^ M, C) or palosuran (10^−6^ M, D), before and during *h*UII application. Right, bar graphs represent the percent increase of the [Ca^2+^]_c_ during drug perfusion or during the washout period. Percent values were obtained by normalizing signals evoked during and after treatments to the value measured before ligand application. Data are mean ± SEM from 9 to 25 cells. ns, non significant; ** *P*<0.01; *** *P*<0.001 compared with the corresponding control Iso-evoked current. In each type of experiment, three different cells have been selected as representative exemples.

To test whether activation of the UT/[Ca^2+^]_c_ signaling pathway may be closely linked to the modulation of GABA_A_R, changes in [Ca^2+^]_c_ evoked by *h*UII, URP, [Orn^5^]-URP and palosuran were measured by continuous Ca^2+^ fluorescence imaging in CHO-UT. When applied to the bath solution, *h*UII and URP evoked a significant and rapid increase of the amplitude of the baseline [Ca^2+^]_c_ by 322% and 341%, respectively (Figure 5A and 5B). The effect of *h*UII was irreversible, only partially recovering after 32-min washout (Figure 5A), as compared with URP whose effect totally recovered after 16-min washout (Figure 5B). It is also observed that [Orn^5^]-URP (10^−6^ M) behaved as a partial agonist (265% of [Ca^2+^]_c_ increase) but prevented the sustained effect of *h*UII on [Ca^2+^]_c_ (Figure 5C). In contrast, palosuran (10^−6^ M) failed to evoke a [Ca^2+^]_c_ rise and completely blocked the *h*UII-induced [Ca^2+^]_c_ increase (Figure 5D).

### Effect of UT on GABA_A_R pharmacology and gating properties

According to the action of UT on GABA_A_R, we asked the question whether the pharmacology and gating properties of GABA_A_R were affected by UT. Thus, we found that the specific positive allosteric GABA_A_R modulator pentobarbital (10^−5^ M) directly activated a chloride current, and reversibly potentiated the Iso-evoked current by 196.28±12.33% (Figure S1A). In addition, SR95531 (10^−5^ M) and picrotoxin (10^−4^ M) induced attended current inhibition by 76.92±10.35%, and by 56.76±4.33%, respectively (Figure S1A). This indicates that pharmacological characteristics of the GABA_A_R are not altered by the presence of UT. Next, to determine whether activation of UT might modify the conductance and selectivity of the GABA_A_R-channel complex, the current-voltage (I-V) relationship was studied on CHO-UT co-expressing α_2_β_3_γ_2_ GABA_A_R subunits. The voltage-dependence of the cell response to Iso (10^−4^ M) was investigated in the absence or presence of *h*UII and the amplitude of the current was measured at different holding potentials (Figure S1B). Local perfusion of *h*UII (10^−8^ M) decreased the slope of the I–V curve but did not significantly shift the chloride reversal potentials (EqCl^−^, control, 4.84 mV; *h*UII, 3.36 mV), closed to the theoretical EqCl^−^ value calculated from the Nernst equation, based on the external and internal chloride concentrations used during recording (see Materials and Methods section). It is observed that *h*UII-induced inhibition of the Iso-evoked current recorded at −60 mV (26.93±6.35%) and +60 mV (37.06±11.25%) was very similar and did not significantly depend on the holding potential (Figure S1B).

**Figure 6:**
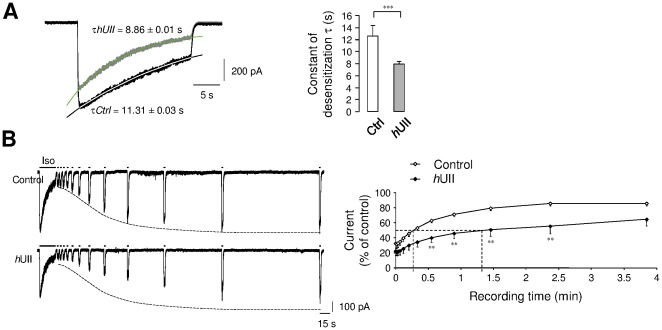
UII-induced fast current inhibition and GABA_A_R desensitization. (A, B) Examples of currents recorded from CHO-UT-GABA_A_R during a long desensitizing pulse (25 s) of Iso (10^−4^ M), in the absence (black line) or presence (green line) of *h*UII (10^−8^ M, 1 min). (A) Exponential fit to the desensitizing current phases were shown overlaid on the currents. Bar graphs corresponding to the average desensitization constant parameter τ in the absence (τCtrl) or presence (τ*h*UII) of *h*UII (n = 5). (B) Prolonged Iso (30 s) application eliciting current desenzitization followed by a time course of the recovery from desensitization, in the absence (control) or presence of *h*UII. Graph represents the Iso-evoked current expressed as a fraction of the peak control current induced by the long Iso application to the current amplitude elicited by each short pulse, and plotted against interpulse intervals. Data are mean ± SEM from 3 to 8 cells. *, *P*<0.05; ** *P*<0.01; *** *P*<0.001 compared with the corresponding control Iso-evoked current.

The subunit composition determines the GABA sensitivity and the pharmacological properties of the GABA_A_R [9] as well as the time course of the GABA response referred as desensitization and deactivation of chloride current [58], [59], [60]. To clearly assess the mechanism of UT-mediating inhibitions of the GABA current, *h*UII was applied on CHO-UT-GABA_A_R on the fast component of current desensitization. As shown in Figure 6, the current evoked by Iso showed a slow decay during continuous agonist ejection as observed by an apparent desensitization of 59% in control, and 77% under *h*UII perfusion. We then followed the time-dependent recovery from desensitization in the absence or presence of *h*UII. Recovering of 50% receptors from this long exposure occurred with mean time constants of 16.2 s in control and 82.2 s in the presence of *h*UII, demonstrating that the progressive recovery is delayed in the presence of the peptide (Figure 6). Thus, when coexpressed with UT, the pharmacological profile of GABA_A_R is not altered, but UII rather affects macroscopic α_2_β_3_γ_2L_ receptor current desensitization, and markedly slows the recovering process. Since recovery from desensitization does not involved membrane voltage [60], it is suggested that UT-mediating GABA_A_R desensitization is mainly due to conformational changes of the ligand-bound receptor chloride channel, paralleled to a mechanism known to develop from the closed but fully bound conducting state of the receptor [61].

### Mechanisms promoting fast short-term and long-term UII-induced GABA_A_R current inhibition

**Figure 7:**
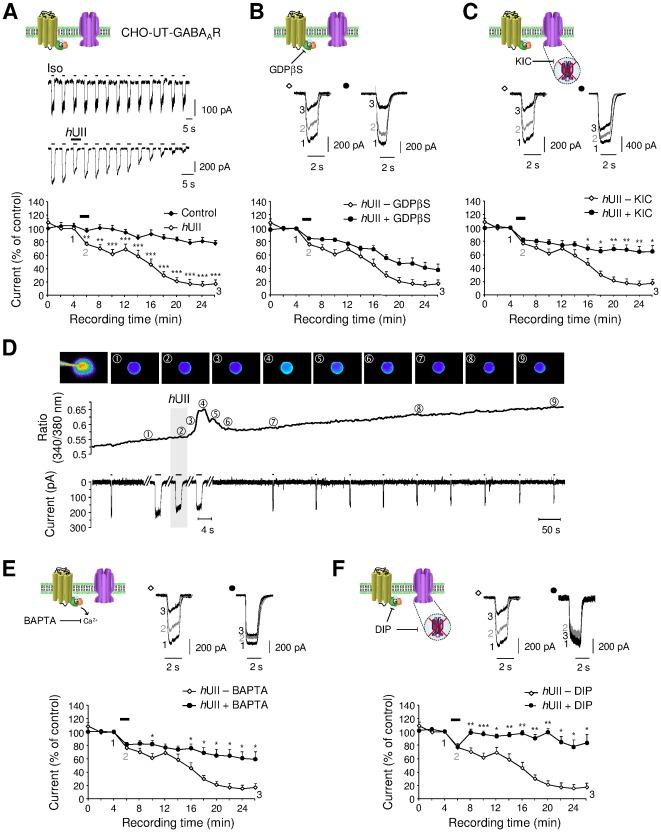
Intracellular mechanisms of UT-triggering GABA_A_R inhibition. (**A**) Traces of Iso (10^−4^ M, 2 s)-evoked current amplitude time-course on CHO-UT-GABA_A_R, in control (above row) or during a 1-min application of *h*UII (10^−8^ M, bottom row). Corresponding average time course of the Iso-evoked current, in control or during and after *h*UII application. (B, C) Current traces before (1), during (2) a 1-min *h*UII application and after 20-min washout (3), in the absence or presence of GDPβS (B, 10^−4^ M, 15 min of dialysis) or the cocktail of kinase and phosphatase inhibitors (C, KIC, 15 min of dialysis). Note that the KIC composition consists in phosphatase inhibitor cocktail at 5 mg/ml (sodium vanadate, sodium molibdate, sodium tartrate and imidazole), Quercetin (10 µM) and staurosporine (10 µM). In the bottom rows are represented the corresponding average time course of the Iso-evoked current, in the absence or presence of GDPβS (B) or KIC (C). (D) Representative [Ca^2+^]_c_ (Fura-2 AM) imaging field before, during *h*UII application and during washout, and time-course of the fluorescence ratio 340/380. Numbers above each curve indicate the corresponding fluorescent image. The bottom row shows simultaneous currents evoked by repetitive Iso ejections, the time scale has been enlarged to show that the current inhibition occurs before *h*UII-induced [Ca^2+^]_c_ rise. (E, F) Current traces before (1), during (2) a 1-min *h*UII application and after 20 min washout (3), in the absence or presence of the rapid Ca^2+^ chelator BAPTA (10^−3^ M, E) or the dynamin inhibitory peptide DIP (10^−5^ M, F). In the bottom rows are represented the corresponding average time course of the Iso-evoked current, in the absence or presence of BAPTA (E) or DIP (F). Data are mean ± SEM from 3 to 21 cells. *, *P*<0.05; ** *P*<0.01; *** *P*<0.001 compared with the corresponding control Iso-evoked current.

In CHO-UT-GABA_A_R, the Iso-activated currents were measured during a 28-min recording period from the initial application of the GABA_A_R agonist. A 1-min application of *h*UII (10^−8^ M) provoked a fast and significant decrease of the current (23.44±2.47%, *n* = 10) followed by a progressive run-down, reaching 84.61±5.92% (*n* = 4) inhibition after 24-min washout (Figure 7A). The second large intracellular loop of several GABA_A_R subunits possesses numerous consensus phosphorylation sites [62] and effective phosphorylation mechanisms have been shown to be involved in either short- or long-term regulation of inhibitory synaptic transmission [63], [64]. To further explore the mechanisms sustaining the *h*UII-induced current long-term depression, we hypothesized that various UT-couplings to G proteins, relay [Ca^2+^]_c_ rise resulting in kinase or phosphatase activation, likely responsible for changes in GABA_A_R subunits phosphorylation state. G-protein activation requires the exchange of bound GDP (resting state) with GTP (activated state). This is a common and necessary step of all G-protein mediated actions and is independent of G-protein type or the second messenger system involved. Therefore, blockade of this exchange will result in inability of the ligand-bound receptor to exert its action. Thus, G protein specific blockade with GDPβS (10^−4^ M) did not significantly prevent the fast and long-term inhibition of the current induced by *h*UII (Figure 7B). Intracellular dialysis with a cocktail of kinase and phosphatase inhibitors (KIC; phosphatase inhibitor cocktail, quercetin 10^−5^ M and staurosporine 10^−5^ M), failed to alter the fast *h*UII inhibitory effect but attenuated the run-down phenomenon (Figure 7C). It is also observed that *h*UII reduced the peak current amplitude after 5-s perfusion, but evoked a peak [Ca^2+^]c increase only after a 10-s delay (Figure 7D). Consistent with this observation, intracellular BAPTA (10^−4^ M) dialysis reduced the long-term current inhibition by only 39.29±10.16% (*n* = 9) (Figure 7E). Together, these observations tend to show that G proteins do not transduce UT-induced current inhibition and that calcium transient and phosphorylation mechanisms do not play a promoting role, but participate in the run-down of the GABA_A_R current. To test a hypothetical role of UII in the dynamin-dependent GABA_A_R endocytosis, the dynamin inhibitory peptide DIP, which competitively blocks binding of dynamin to amphiphysin [64], has been introduced in the intrapipette solution. As shown in Figure 7F, when cells were dialyzed with DIP (10^−5^ M), *h*UII retained its ability to induce a fast and highly reversible inhibition of the Iso-evoked current, but failed to reduce the current amplitude with time recording.

### Inhibition of the GABAergic activity involves GABA_A_R internalization and requires specific UT receptor domains in CHO and human astrocytes

**Figure 8:**
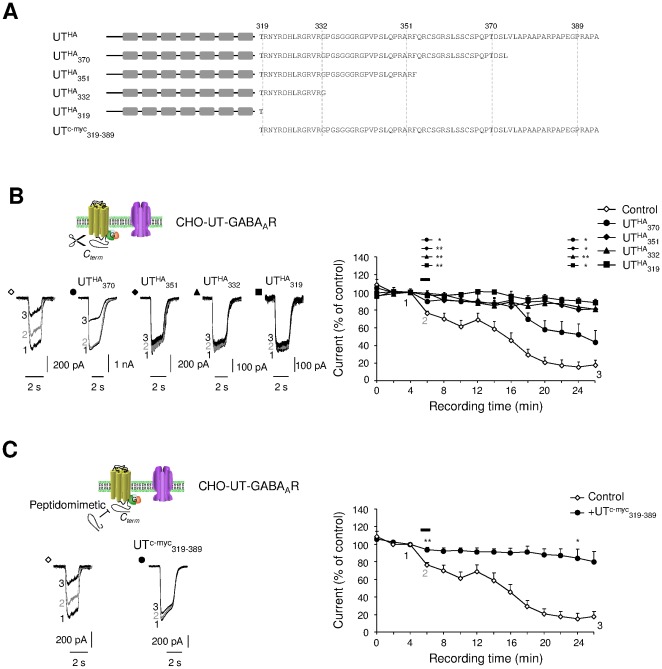
Receptor sequences involved in UT regulation of the GABA_A_R activity. (A) Schematic diagrams mixed with sequence alignments of the HA epitope-tagged human UT, C-terminus truncated UT^HA^_370_, UT^HA^_351_, UT^HA^_332_, UT^HA^_319_ mutants, and peptidomimetics corresponding to the entire C-terminus cytosolic fragment of UT (UT^c-myc^_319–389_). (B and C) Traces of Iso (10^–4^ M, 2 s)-evoked current before (1), during (2) a 1-min *h*UII (10^−8^ M) application and after 22-min washout (3). (B) Currents recorded from CHO coexpressing GABA_A_R and UT^HA^ (Control), UT^HA^_370_, UT^HA^_351_, UT^HA^_332_ or UT^HA^_319_. Corresponding average time course of the current, in the absence or presence of UT truncated mutants. (C) Current traces recorded from CHO-UT-GABA_A_R, in the absence or presence of UT^c-myc^_319–389_. Corresponding average time course of the Iso-evoked current, in the absence or presence of UT^c-myc^_319–389_. In B, significance was only annotated above the time course graph during *h*UII perfusion and after 18-min washout, for clarity. Data are mean ± SEM from 3 to 13 cells. ns, non significant; *, *P*<0.05; ** *P*<0.01 compared with the corresponding control Iso-evoked current.

To assess whether the C-terminus fragment of UT, corresponding to the cytoplasmic C-tail of the receptor contributes to the UII-induced run-down of the GABA_A_R activity, we constructed four truncated mutants in which the last 19 (UT^HA^_370_), 38 (UT^HA^_351_), 57 (UT^HA^_332_) or 70 (UT^HA^_319_) residues were removed (Figure 8A). They are all present at the plasma membrane (Figure S2) and functionally expressed except the UT^HA^_319_ truncated form of UT (Figure S3). When UT^HA^_370_ is coexpressed with GABA_A_R, the *h*UII-induced fast short-term inhibition was totally abolished, whereas the long-term inhibition was delayed, and significantly altered (Figure 8B). In the presence of shorter truncated forms, *h*UII totally failed to alter the GABA_A_R activity, suggesting that the most distal C-terminus part of UT is involved in the functional cross-talk with GABA_A_R (Figure 8B). Moreover, the peptidomimetic UT^c-myc^_319–389_ (Figure 8A) completely inhibited the fast and long-term effects of *h*UII on the GABA-evoked current (Figure 8C). Collectively, these results indicate that the C-terminus of UT relays the inhibition of the GABA_A_R function, and may counteract a potential *h*UII-induced internalization process.

**Figure 9:**
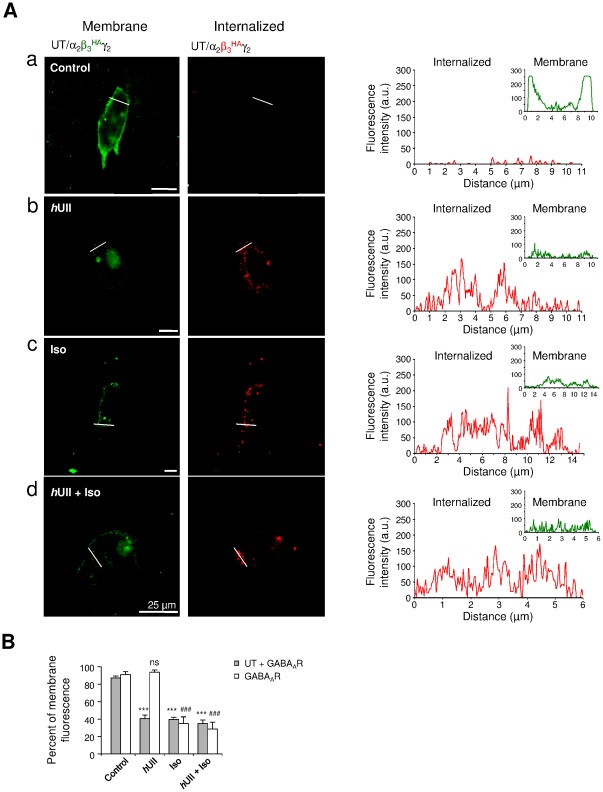
UT activation mediating GABA_A_R internalization. (Aa–Ad) CHO-UT transiently transfected with cDNA encoding α_2_β_3_^HA^γ_2_ GABA_A_R subunits. Internalization was controlled through translocation of β_3_^HA^ subunit (red) in control (Aa) or after 60 min of *h*UII (10^−8^ M, Ab), Iso (10^−4^ M, Ac) or *h*UII+Iso (Ad) incubation. Fluorescence intensity plots of green and red fluorescences corresponding to the localization of GABA_A_R (β_3_^HA^) at the plasma membrane and in the cytosol, respectively, across the regions delimited by the white line scans. A.u., arbitrary unit; scale bars, 25 µm. (B) Bar graphs of the fraction of fluorescence at the plasma membrane on CHOT-UT-GABA_A_R or CHO-GABA_A_R in the different conditions. Each bar corresponds to mean ± SEM percent obtained from 3 to 18 cells. ns, non significant; ***, *P*<0.001 *versus* control in CHO-UT-GABA_A_R; ###, *P*<0.001 *versus* control in CHO-GABA_A_R.

Our data thus suggest that UT activation likely regulates GABA_A_R endocytosis. We first established the subcellular localization of both UT and GABA_A_R in cultured CHO transiently transfected with cDNAs encoding recombinant human UT and the α_2_β_3_^HA^-γ_2_-tagged (α_2_β_3_^HA^γ_2_) GABA_A_R subunits and then, internalization of GABA_A_R was followed by labeling the surface receptors with antibodies directed against the β_3_^HA^ co-expressed with α_2_γ_2_ GABA_A_R subunits and/or UT. In the absence of ligand, the immunoreactivity for β_3_ exhibited membrane localization (green) as enlighted by the intensity profiles (Figure 9Aa). In contrast, treatment with *h*UII (10^−8^ M), Iso (10^−4^ M) or the two agonists, drastically promoted GABA_A_R endocytosis by 40.29±4.14%, 39.31±2.84% and 34.71±3.19%, respectively (Figure 9B), as seen by the increase of red punctuates in the cell soma (Figure 9Ab-8Ad). When GABA_A_R was expressed alone, *h*UII failed to induce GABA_A_R internalization whereas Iso or Iso combined with *h*UII remained able to provoke GABA_A_R removal from the plasma membrane (Figure 9B).

**Figure 10:**
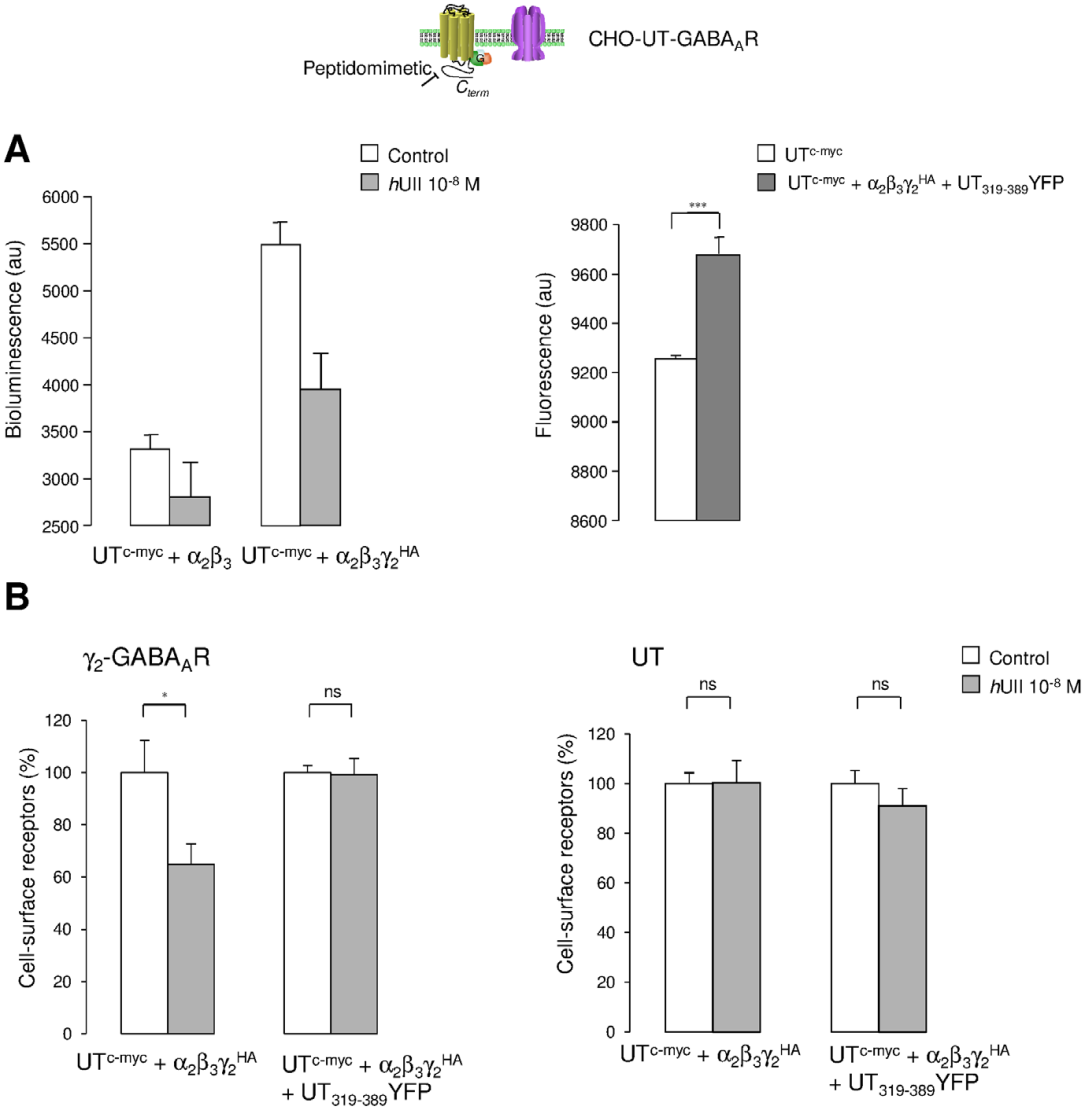
UII-induced GABA_A_R loss from the plasma membrane through the C-terminus fragment of UT in CHO. The effect of *h*UII on the proportion of GABA_A_R and UT at the cell surface of CHO was assessed by ELISA. (A) CHO transiently transfected with cDNA encoding UT^c-myc^ and α_2_β_3_, or α_2_β_3_γ_2_^HA^ GABA_A_R subunits (left), or UT^c-myc^, and α_2_β_3_γ_2_^HA^ GABA_A_R subunits cotransfected with the cDNA encoding UT_319–389_YFP (right). Background bioluminescence (left) and fluorescence (right) were measured after anti-HA antibody and colorimetric alkaline phophatase substrate incubation, in the absence or presence of 30 min of *h*UII (10^−8^ M, left), or directly on a fluorescent plate reader (right). (B) CHO transiently transfected with cDNA encoding UT^c-myc^ and α_2_β_3_γ_2_^HA^ GABA_A_R subunits (left), or cotransfected with the cDNA encoding UT_319–389_YFP, and immunodetected with anti-HA (left) or anti-c-myc (right) antibodies. Percentage of cell surface γ_2_^HA^ GABA_A_R subunit (left) or UT^c-myc^ (right) are represented as the proportion of receptor at the plasma membrane (non permeabilized cells) to the total expressed receptor (permeabilized cells). One hundred percent correspond to values in the absence of 30 min treatment with *h*UII (10^−8^ M, 37°C). Each bar corresponds to mean ± SEM percent obtained from 5 to 7 independent experiments, in triplicates. ns, non significant; *, *P*<0.05; ***, *P*<0.001.

**Figure 11:**
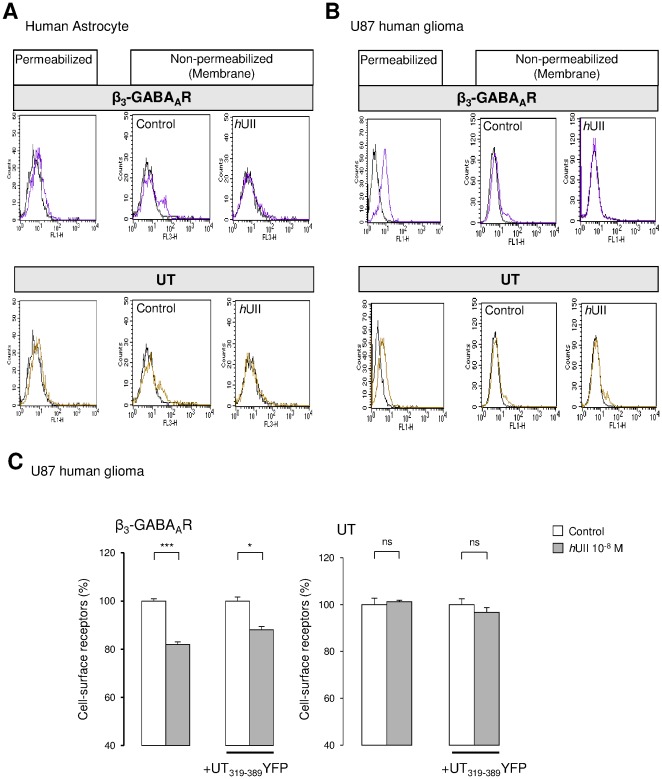
UII-evoked GABA_A_R internalization in native human astrocytes and glioma. (A, B) FIow cytometric analysis of the β_3_ GABA_A_R subunit and UT expression in native human astrocytes (A) and human U87 glioma cell line (B). Cells were stained with the anti-human β_3_ subunit or anti-human UT in permeabilized or non permeabilized conditions (membrane receptor). The black lines depict results from control staining with only secondary antibodies. The β_3_ GABA_A_R subunit or UT cell surface expression was evaluated in the absence or presence of *h*UII (10^−8^ M, 30 min) by flow cytometry. Data obtained in A and B illustrate two representative experiments showing β_3_ (magenta line) and UT (yellow line) mean fluorescence in the cytosol and at the plasma membrane of a minority of non permeabilized human astrocytes (A) or U87 (B) in culture. The exposure to *h*UII induced internalization of β_3_ in both cell types and of UT only in U87 glioma. (C) U87 glioma cell line expressing UT and GABA_A_R composed of β_3_ subunit, and transfected with the cDNA encoding UT_319–389_YFP, and immunodetected with anti-β_3_ (left) or anti-UT (right) antibodies. Percentage of cell surface β_3_ subunit (left) or UT (right) are represented as the proportion of receptor at the plasma membrane (non permeabilized cells) to the total expressed receptor (permeabilized cells). One hundred percent correspond to values in the absence of 30 min treatment with *h*UII (10^−8^ M, 37°C). Each bar corresponds to mean ± SEM percent obtained from at least 3 independent experiments, in triplicates. ns, non significant; *, *P*<0.05; ***, *P*<0.001.

In order to confirm the involvement of UII/UT in the internalization process of GABA_A_R in CHO, we investigated the plasma membrane expression of the γ_2_^HA^ GABA_A_R subunit, as well as UT^c-myc^ after exposure to *h*UII by measuring the amount of surface immunolabelled receptors by ELISA. Typical bioluminescence and fluorescence values obtained from CHO expressing either UT^c-myc^-α_2_β_3_ or UT^c-myc^-α_2_β_3_γ_2_^HA^, and UT^c-myc^ or UT^c-myc^-α_2_β_3_γ_2_^HA^-UT_319–389_YFP, respectively, were shown in Figure 10A. In CHO-UT^c-myc^-α_2_β_3_γ_2_^HA^, a 30 min treatment with *h*UII (10^−8^ M) led to approximately 40% loss of γ_2_^HA^ subunit from the cell surface, without modifying cell membrane amounts of UT^c-myc^ (Figure 10B). In CHO-UT^c-myc^-α_2_β_3_γ_2_^HA^ cotransfected with the cDNA encoding the UT_319–389_YFP peptidomimetic fragment, *h*UII incubation failed to remove the γ_2_^HA^ subunit from the plasma membrane (Figure 10B), establishing that the C-terminus part of UT played a major role in the UII-induced GABA_A_R internalization in a recombinant system. The physiological relevance of such mechanism was thus assessed in native human astrocytes and in the human glioma U87 cell line. Flow cytometry analysis showed that normal and tumoral glial cells in culture expressed β_3_ subunit (Figure 11A) and UT (Figure 11B), and that one population (around 8%) of astrocytes and U87 exhibited both receptors at the plasma membrane (Figure 11). *h*UII (10^−8^ M, 30 min) provoked more than 65% GABA_A_R disappearance from the surface, whereas UT internalized in native astrocytes (Figure 11A) but was still present at the cell surface in U87 (Figure 11B). We then examined the cell surface stability of GABA_A_R following UT activation in the absence or presence of the coexpressed UT_319–389_YFP on U87 glioma cell line. ELISA-based assay revealed that the UT_319–389_ fragment reduced the β_3_-associated GABA_A_R subunit internalization from 20% to approximately 10% (Figure 11C), with an efficacy of transfection of around 32%, as controlled by flow cytometry (*data not shown*). As already shown in CHO, UT levels at the cell surface remained unchanged after exposition to *h*UII, in the absence or presence of the UT C-terminus (Figure 11C).

## Discussion

The question of the regulation of the GABA_A_R plasticity and cross-modulation in extrasynaptic glial cells by neurovascular factors, and its functional consequence were not explored. In rodent brain, mRNA encoding the vasoactive receptor UT are detected in the olfactory system, hippocampus, amygdala, tegmental nuclei, or cerebellum [27], [65], [66] and UII binding sites are restricted to few areas including the cerebellar cortex [39], [67]. We and others have also shown that UT receptor expression may be specific to glial cells *in vivo* [40], and to astrocytes in culture [41], as well as to brain microvascular entities (*unpublished data*).

In the present study, to gain insight into the regulation of the GABAergic activity by the vasoactive peptide UII in astrocyte, the cellular and subcellular distribution and feature of the UT receptor, have been investigated in the cerebellum in situ, and in astrocyte-neuron co-cultures. We showed intense UT labeling in Purkinje cell bodies and ramifications as well as in glial Bergmann-GFAP positive long processes where it co-localizes with γ_1_ and, to a lesser extent, with γ_2_ subunits of the GABA_A_R, and in astrocytes co-cultured with granule neurons. Together, this corroborates previous studies establishing expression of α_5_, γ_1_ and γ_3_ subunits in the PCL [68], [69], and of γ_1_ mRNA in Bergmann glia [70], [71]. We confirmed the coexpression of GABA_A_R subunits with UT in native human astrocytes and in the U87 glioma cell line. It is interesting to note that a majority of glial cells expresses at least the β_3_ GABA_A_R subunit and also UT in the cytosolic compartments, but that only around 10% of cells corresponding to a common subpopulation, show these receptors at the plasma membrane. This is in a good agreement with the 12% responding rat cultured astrocytes to isoguvacine in mono-culture. In fact, the existence of two populations of astrocytes, exhibiting depolarized membrane potentials (around −30 mV) in a majority of cells and hyperpolarized membrane potentials (around −80 mV) in a minority (*data not shown*) was observed in our study, as already shown in cultured astrocytes [72], independent on patch-clamp recording conditions. Here, we determined that this hyperpolarized subpopulation represents astrocytes specifically responding to the GABA_A_R activation. Since it was suggested that GABA acts as an anti-proliferating neurotransmitter in ventricular and subventricular zones [73] and in cortical progenitor cells [74] and that down regulation of functional GABA_A_R is correlated with the proliferzation rate of reactive or malignant astrocytes [15], [25], we propose that rat and human cell subpopulation expressing GABA_A_R likely correspond to quiescent astrocytes in culture.

These colocalized features of UT and GABA_A_R in astrocytes prompted us to investigate a potential functional cross regulation between the two receptors, likely involved in astrocyte plasticity. It has been shown that astrocytic GABA response is specific of early culture period which is maintained by interaction with neurons [75]. We demonstrate that UII down regulates the Iso-evoked depolarization and chloride current amplitudes recorded from astrocytes co-cultured with granule neurons. This UII-induced GABA_A_R current inhibition is shown to be a very high affinity process, specific of astrocytes, which hardly recovered during washout. In CHO co-expressing human UT and αβ and/or γ GABA_A_R subunits, we found that UII was *i)* very potent on β_1/3_γ_1/2L_ GABA_A_R subunit complexes, *ii)* less potent on α_2_β_1/3_γ_1/2L_ complexes and *iii)* inactive on αβ binary complexes. These results thus establish that UII exhibits a very high affinity directional inhibition toward the GABA_A_R specifically composed of the γ subunit. This observation can be paralleled to the high affinity binding sites for UII determined on recombinant UT-expressing cells [76], [77], [78], [79], and also on astrocytes [41]. The time course of the UT-evoked current inhibition can be distinguished by two phases, *i.e.* a short-term decrease detected immediately after and during UII administration, followed by a progressive run-down of the current, leading to about 80% GABA_A_R current disappearance. However, URP which exhibits the same conserved biologically active cyclic sequence than UII, triggers a reversible inhibition. This is in accordance with the UII-induced long-lasting in the one hand, and the URP-evoked transient, on the other hand, increase in [Ca^2+^]_c_ in CHO-UT. The specific long-lasting phenomenon might be attributable to the slow dissociation rate of UII, as already described for rat and human UII on UT transfected cells, skeletal muscle myoblasts and astrocytes [41], [54], [79]. This could account for the sustained and washout-resistant contractile responses induced by UII on primate arteries, [Ca^2+^]_c_ increase in rat cortical astrocytes [42], and GABA_A_R current inhibition in our native and recombinant systems. We propose a mechanism whereby UII interacts reversibly with the classical binding site, but also with a secondary exosite in a wash resistant manner, resulting in persistent activation of UT and consequently, in a long-term inhibition of the GABA_A_R. Such process has already been demonstrated *in vivo* and cell culture, for exogenous agonists of M1 muscarinic and β_2_ adrenergic receptors [80], [81]. Together, our previous work suggesting that GABA negatively controls UT-mediating signaling transduction in astrocytes [43], corroborated by the effect of benzodiazepines on UII-induced neurotransmitter release [82], supports at most the existence of a negative cross-talk coupling between UT and the closely associated GABA_A_R, leading to a high affinity functional receptor complex in astrocytes. This functional complex may exhibit new pharmacological profile. Accordingly, we demonstrated that [Orn^5^]-URP acts as a partial agonist and competitive antagonist on both GABA currents and [Ca^2+^]_c_. Surprisingly, palosuran as a specific primate UT antagonist [55], counteracts the UII-evoked [Ca^2+^]_c_ increase but mimics the effect of UII by inhibiting the chloride current. Thus, palosuran behaving as an antagonist of the UT-mediating [Ca^2+^]_c_ transduction signaling can be considered as a partial UT “agonist" toward the GABA_A_R effector pathway, then suggesting a different UT pharmacology when co-expressed with GABA_A_R.

Here we found that initiation of UT and GABA_A_R functional interaction is independent on G protein, calcium and phosphorylation mechanisms, but that UII-induced current run-down partially requires calcium and kinase/phosphatase activities. In this context, the Ca^2+^/calmodulin requirement for membrane fusion in endocytic pathways [83], as well as the clathrin-mediated internalization depending on kinase and phosphatase activities[84], [85], [86], support here a possible role of UT in a delayed calcium/kinase dependent GABA_A_R endocytosis. GABA_A_R internalization is primarily thought to occur *via* a clathrin- and dynamin-dependent mechanism [87]. Here DIP, known to block endocytosis by disrupting the interaction between dynamin and amphiphysin, did not interfere with the UII-induced fast short-term but totally abolished the long-term inhibition of the current, supporting a role of UT in the dynamin-dependent GABA_A_R internalization. We then show that the GABA_A_R agonist triggered removal of β_3_ or γ_2_ GABA_A_R subunit from CHO plasma membrane, or from native human astrocytic and glioma cell surface. Together, the interesting point resides in the ability of the UII/UT system in the promotion of the marked GABA_A_R internalization in the absence of co-activation of GABA_A_R. In addition, 30 min incubation with UII failed to internalize UT in CHO and U87, but led to UT loss from human astrocyte surface, a discrepancy unexplained but needing further investigations. Thus, a constitutive tight promiscuity between UT and GABA_A_R might be responsible for the high affinity effect of UII on GABA_A_R disappearance from the plasma membrane.

Here we produced and expressed truncated UT receptors in order to identify which specific receptor determinants are involved in the GABA_A_R modulation. The deletion of up to 57 residues of the UT C-terminus, did not affect the expression and ability of truncated mutants to stimulate the [Ca^2+^]_c_, as already observed for rat UT truncated mutants [88]. We demonstrate that the entire UT C-terminus totally abolished the UII-mediating current inhibition and that the most distal part likely relays the fast and long-term inhibitions of the GABA_A_R function. In rat, the last 19 residues of the C-terminus contain motifs that are not crucial for UT internalization [88]. In fact, the serine cluster localized upstream rather displays consensus motifs for PKC and casein kinase I important for rat UT internalization. In humans, the distal UT C-terminus (351–389) exhibits serine residues and a combination of two polyproline motifs (Figure 8A), the last one possibly interacts with SH3 domain proteins [89]. Our present data in CHO co-expressing human UT and α_2_β_3_ and γ_2_ GABA_A_R and in U87 transfected with the cDNA encoding the UT_319–389_ peptidomimetic, indicate that the UII-induced internalization of GABA_A_R formed from γ_2_ or β_3_ subunit, requires at least in part, the C-terminus fragment of UT. Therefore, the question of whether UT and GABA_A_R physically associate directly or whether SH3 proteins relayed GABA_A_R down regulation, has to be elucidated. Together, the functional cross-modulation between UT and GABA_A_R is mediated through the most distal part of the UT C-terminus, which would directly interact with γ subunits, or recruit intermediate proteins implicated in GABA_A_R inhibitory transactivation.

**Figure 12:**
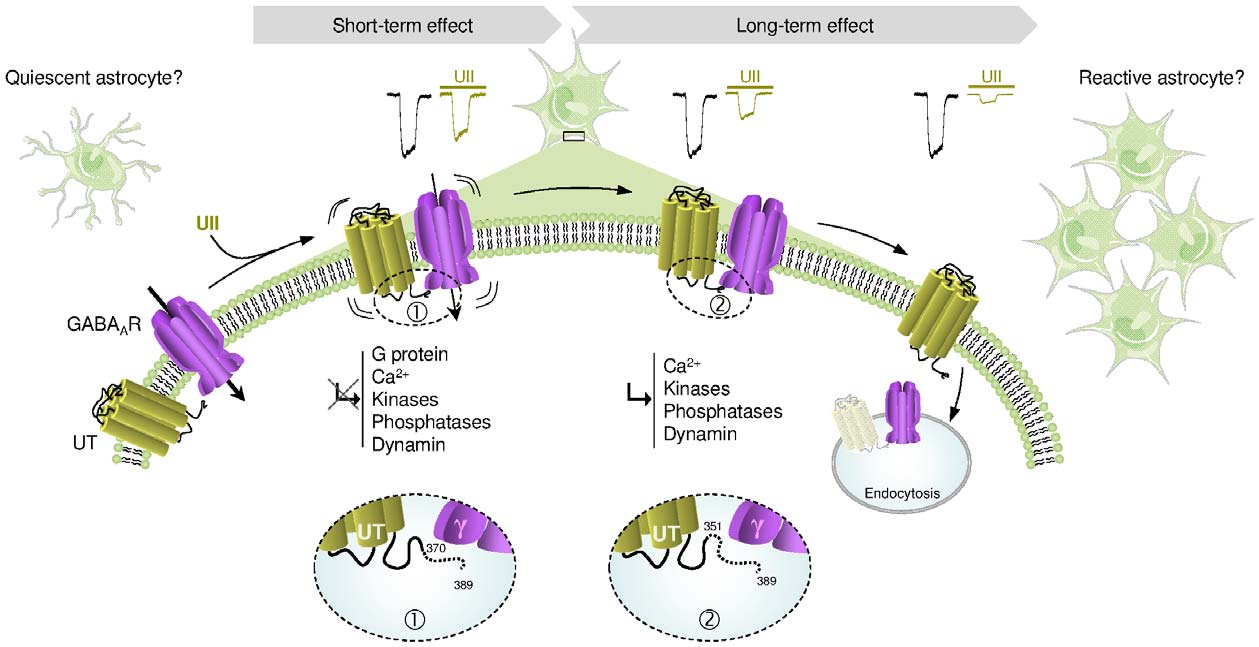
Schematic model depicting the mechanism of UT-mediated GABA_A_R down-regulation. UII efficiently activates the G protein-coupled receptor UT, leading to a fast short-term decrease of the chloride current not sustained by G proteins, calcium, phosphorylation and endocytosis processes. This rapid effect involves the distal 19 C-terminal amino acids of UT and the presence of γ subunits within of the GABA_A_R complex (1). During the washout period, a long-term inhibition develops *via* a dynamin-, calcium- and phosphorylation-dependent endocytic mechanisms, requiring at least in part the 351–370 sequence of UT and GABA_A_R γ subunits (2). It is hypothesized that the directional cross-talk between UT and GABA_A_R, and the extinction of the latter at the plasma membrane, may relay transition from quiescent to proliferant astrocytes.

Therefore, our observations suggest a model in which UT and GABA_A_R are closely associated to depress the GABAergic activity (Figure 12). When UT and αβγ GABA_A_R subunits are co-expressed, as in native and tumoral glial cells, UII efficiently activates its receptor, leading to a fast short-term decrease of the chloride current, independently of G proteins, calcium, phosphorylation and endocytosis processes, and involving the last 19 amino acids of the UT C-terminus. During washout, a long-term inhibition referred as run-down, develops *via* a dynamin-dependent internalization requiring the 351–370 sequence of UT, and calcium- and phosphorylation-dependent endocytic mechanisms. This directional cross-talk between UT and the GABA_A_R leads to the extinction of the GABA_A_R expression at the plasma membrane that would play a key role in the induction of cell proliferation (Figure 12).

## Materials and Methods

### Animals

Wistar rats (Depré, Saint-Doulchard, France) were kept in a temperature-controlled room (21±1°C), under an established photoperiod (lights on 07.00–19.00 h) with free access to food and tap water. The work described in this article was carried out in accordance with the Directive 2010/63/EU of the european parliament and of the council of 22^th^ September 2010 on the protection of animals used for scientific purposes, published in the Official Journal of the European Union L276/33 (20.10.2010) and authorized by the French Ethical Committee. These experiments were conducted under the supervision of authorized investigators (H. Castel; authorization no. 76.98 from the Ministère de l'Alimentation, de l'Agriculture et de la Pêche) and were approuved by the local animal ethic committee of Normandy, approuval number N/02-09-09/03/09-12.

### Primary Cell Culture of astrocytes and astrocyte-neurone co-culture

Primary cultures of astrocytes were prepared as previously described [90]. Briefly, cerebellum from 7-day-old (P7) Wistar rats were collected in DMEM/Ham-F12 (2∶1, v/v) culture medium supplemented with 2 mM glutamine, 1% insulin, 5 mM HEPES, 0.4% D(+)-glucose and 1% of the antibiotic-antimycotic solution. The tissues were disaggregated mechanically with a syringue equipped with a 1-mm gauge needle, and filtered through a 100-µm pore size mesh filter (Falcon, Becton Dickinson, Grenoble, France). Dissociated cells were resuspended in culture medium supplemented with 10% heat-inactivated FBS and seeded in 150-cm^2^ culture flasks (Falcon) at a density of 20×10^6^ cells/flask. Cells were incubated at 37°C in a humidified atmosphere (5% CO_2_) and the medium was changed twice a week. When cultures were confluent, the flasks were gently shaken on an orbital shaker at 250 g for 2 h. Dislodged cells were discarded and a second step of purification was performed at 250 g for 14–16 h. Remaining adhesive cells were collected by trypsination, centrifuged (800 g, 10 min) and plated in 150-cm^2^ flasks. Suspended astrocytes were harvested and seeded in 24-well poly-L-lysine-coated plates. The purity of the cultures was previously assessed by counting the percentage of astrocytes immunostained with GFAP antibodies. The enriched cultures contained >99% astrocytes [41].

For astrocyte-neuron co-culture, granule cell cultures were prepared from cerebella of P7 Wistar rats as described previously [91]. Isolated cells were plated on 14-mm culture dishes coated with poly-L-lysine (5 mM) at a density of 1.5×10^6^ cells/dish and incubated at 37°C in a humidified atmosphere (5% CO_2_) for 1 to 10 days before use. Culture medium consisted of DMEM/Ham's F12 (75%/25%) supplemented with 10% FBS, 2 mM glutamine, 5 µg/ml insulin, 25 mM KCl and 1% of antibiotic-antimycotic solution. Co-cultures are obtained by seeding granule cells (1.5×10^6^ cells/ml) on cerebellar astrocytes plated in 24-well plates after 12 hours. Co-cultures are maintained in the specific medium for neuron and incubated at 37°C in an humidified atmosphere (5% CO_2_) for several days.

### Human native astrocytes and glioma cell line

The human cell line from glioblastoma U87 was obtained from the American Type Culture Collection (LGC Standards, Molsheim, France). U87 cells were maintained in DMEM containing 10% FBS and 1% sodium pyruvate. NHA-Astrocytes (Lonza, Walkersville, MD, USA) were cultivated in DMEM culture medium supplemented with 2 mM glutamine, 5 mM HEPES, 1% non essential amino acids, 1% sodium pyruvate, B27, 25 ng/ml EGF, 1% of the antibiotic-antimycotic solution and 10% FBS. All cells were incubated at 37°C in a humidified atmosphere containing 5% CO2. Culture media were replaced every three days.

### CHO recombinant cell line and plasmid transfection

CHO-K1 cell lines were obtained from American Type Culture Collection (Manassas, VA, USA). The human UT stable CHO (CHO-UT) was generously provided by Dr Christophe Dubessy (Inserm, Rouen University, France) et generated by CHO electroporation with 20 µg pIRES-neo2-UT DNA and 500 µg sterile sonicated salmon sperm DNA using the EasyJect One electroporation system (Equibio, Angleur, Belgium), followed by repetitive rounds of limiting dilution of cells in G-418 (400 µg/ml) for selection. Normal or stable CHO was grown in Ham-F12 medium supplemented with 10% FBS, 1% antibiotic-antimicotic solution and 2 mM glutamine, at 37°C in a humidified incubator with an atmosphere of 5% CO_2_. The stable CHO-UT medium was supplemented with the antibiotic G-418 (40 µg/ml).

For transfection, cells were trypsinized (0.05%), triturated in HamF-12 containing 10% FBS media, pelleted by centrifugation, resuspended in 100 µl solution V for nucleofection by an Amaxa Nucleofector Device (Köln, Germany; set to program U-016). Experiments were performed on normal CHO or on the stable CHO-UT cells and transiently transfected with combinations of cDNA encoding α_2_β_3_γ_2_, α_2_β_3_γ_1_, α_2_β_1_γ_2_, α_2_β_3_γ_1_, α_2_β_3_, β_3_γ_2_ (4 µg of cDNA total/transfection) GABA_A_R subunits. Cells were seeded on 14-mm poly-L-lysine-coated glass bottom insert dishes at 5×10^5^ cells in a volume of 0.5 ml/dish. Cells were incubated overnight at 37°C in a humidified incubator (5% CO_2_) during 16 h before electrophysiological or immunocytochemical experiments.

### Recombinant receptors

For UT epitope-tagged with HA (UT^HA^), or c-myc (UT^c-myc^), human UT receptor cDNA inserted into pcDNA3.1 (Ressource Center, MI, USA) was amplified and PCR products were subcloned using the EcoRI and XhoI sites of pCMV-HA or pCMV-c-myc. Mutant UT receptor cDNAs were constructed by oligonucleotide-directed mutagenesis (Expand High Fidelity PCR System; Roche) using the human UT receptor cDNA inserted into pcDNA3.1 as a template. Two sets of forward and reverse oligonucleotides were used (Table S2) to introduce stop codons in frame of Leu370, Phe351, Gly332 and Thr319 (to generate UT_371_, UT_351_, UT_332_ and UT_319_, respectively). PCR products were subcloned using the EcoRI and XhoI sites of pCMV-HA after digestion by the same restriction enzymes. Mutagenesis was confirmed by automated nucleotide sequencing.

GABA_A_ receptor cDNA clones; α_2_, β_1_, β_3_, γ_1_ and γ_2L_ engineered into the expression vector pCDM8 (α_1_, β_1_, γ_2_) or pcDNA/Amp (β_3_, γ_1_), were generously provided by Dr Wingrove (Merk Sharp and Dohme, Harlow, UK). To obtain β_3_^HA^ or γ_2_^HA^ epitope-tagged subunits, β_3_ or γ_2_ cDNA was amplified and the PCR product was subcloned using the SalI and NotI sites of pCMV-HA (Table S2) after digestion by the same restriction enzymes.

In order to generate mini-peptides corresponding to the C-terminus of UT, cDNA encoding the UT^c-myc^_319–389_ or UT_319–389_YFP fragment was amplified by PCR. The 5′ and 3′ oligonucleotides incorporated SalI and NotI or EcoRI and BamHI sites, respectively, to facilitate subcloning into pCMV-c-myc or pEYFP-N1 (BD Biosciences Clontech, Mississauga, ON, Canada), and incorporated initiation and stop codons where appropriate (Table S2).

### Immunocytochemistry on co-culture

Astrocyte-neuron co-cultured on glass coverslips were washed three times in PBS, fixed in 4% paraformaldehyde at 4°C for 20 min, and washed three times in PBS. Cells were permeabilized in PBS containing 0.1% Triton X-100 (10 min) and pre-incubated with normal goat and/or normal donkey antiserum (1∶50, Santa-Cruz, Tebu bio, Le Perray en Yvelines, France) for 1 h. Then, cells were incubated at 20°C for 1 h with a goat anti-UT (1∶200), a mouse anti-NeuN (1∶200, Santa-Cruz) or a rabbit anti-GFAP (1∶1000, Dako, Trappes, France). Specificity of the UT immunolabelling on astrocytes has already been demonstrated [40]. After several rinses in PBS, cells were incubated at 20°C for 2 h with Alexa 488-conjugated donkey anti-goat and anti-Alexa 594-conjugated donkey anti-rabbit IgGs diluted 1∶300 (Invitrogen, Boulogne Billancourt, France).

### Receptor cell surface internalization

For double-immunofluorescence, non-permeabilized living CHO cells expressing UT and/or α_2_β_3_^HA^γ_2_ GABA_A_ receptors were washed two times in PBS, and incubated with monoclonal mouse anti-c-myc and rabbit polyclonal anti-HA (1∶200, Santa-cruz) for 1 h on ice in DMEM. Excess antibody was removed and cells were incubated with the different receptor agonists for 30 min at RT. After several rinses in PBS, cells were fixed in 4% paraformaldehyde at 4°C for 20 min, washed three times in PBS and then incubated at RT for 2 h with appropriate secondary antibodies, *i.e.* Alexa-488- and 594-conjugated donkey anti-mouse IgGs diluted 1∶300 (Invitrogen). After washing, coverslips were mounted in Eukitt (VWR International, Strasbourg, France).

All preparations were examined using a confocal laser-scanning microscope (Leica, Heidelberg, Germany) equipped with a Diaplan optical system, a UV laser (excitation wavelength 405 nm) and argon/krypton ion (excitation wavelengths 488/594 nm) laser.

### Immunohistochemistry on cerebellar sections

Following decapitation, the cerebellum of 7-day-old (P7) Wistar rats was extracted and immersed in iced PBS. Cerebella of P7 rats were sectioned transversely into 180 µm-thick slices on a vibrating blade microtome (VT1000S, Leica Instruments). The slices were subsequently washed in PBS (pH 7.4) and postfixed in a 4% PFA solution for 20 min. Thereafter, free-floating sections were rinsed and non-specific binding was blocked by 10% normal donkey serum, 0.1% BSA and 0.3% Triton X-100 in PBS for 1 h. The tissue sections were incubated overnight at 4°C with a goat antiserum directed against rat UT (1∶200, Santa Cruz), a mouse anti-calbindin (1∶400, Sigma-Aldrich, Saint-Quentin Fallavier, France), a mouse anti-GFAP (1∶1000, Dako), a mouse anti-NeuN raised in mouse (1∶200, Santa-Cruz), an anti-doublecortin (DCX) raised in goat (1∶400, Santa-Cruz), or anti-γ_1_ and anti-γ_2_ GABA_A_R subunits (1∶200, generous gift from Dr Sieghart, Brain Research Institute, Vienna, Austria). The sections were rinsed three times with PBS and incubated for 2 h at RT with Alexa 488- or 594-conjugated donkey anti-rabbit, donkey anti-goat or donkey anti-mouse (1∶200, Invitrogen). After washing, slices were incubated with 4,6-diamidino-2-phenylindole (DAPI, 1∶10000, Sigma-Aldrich) for 5 min to label nuclei. Finally, the sections were rinsed in PBS, and mounted with mowiol. To study the specificity of UT and other marker inmmunoreactivities, the following controls were performed (1) substitution of each antiserum by PBS, and evaluation of the level of fluorescence given by each type of secondary antibody, (2) systematic mono-immunolabeling of each protein marker. The preparations were examined on a Leica SP2 upright confocal laser scanning microscope (DM RXA-UV) equipped with Acousto-Optical Beam Splitter (AOBS) system. For confocal images, Alexa-488 and Alexa-594 were excited respectively at 488 and 594 nm.

### Electrophysiology

The conventional whole-cell configuration of the patch-clamp technique was used to study the GABA-gated currents in astrocytes and granule neurons, UT stably transfected CHO, CHO-UT, and CHO transiently transfected with diverse variants of UT and GABAAR subunits. After 24-h transfection, cell culture coverslips were placed in a small chamber (1.5 ml) on a stage of a right microscope DMLFSA (Leica, Heidelberg, Germany) and superfused continuously with the following bath solution containing (in mM): NaCl, 150; KCl, 2.5; HEPES, 5; CaCl2, 2; MgCl2, 1; glucose, 10 (pH 7.4 adjusted with NaOH). The patch pipettes were fabricated from 1.5 mm (outer diameter) soft glass tubes on a two-step vertical pipette puller (List-Medical, L/M-3P-A, Darmstadt, Germany). Patch electrodes had a final resistance of 4–6 MΩ when filled with an internal pipette solution containing (in mM): KCl, 130; MgCl2, 2; CaCl2, 0.5; EGTA, 5; HEPES, 10; ATP, 1; GTP, 0.1 (pH 7.4 adjusted with KOH). ATP and GTP were added to the internal solution used to fill electrode just before recording. All recordings were obtained at RT with cells voltage-clamped at −70 mV. The GABA_A_ receptor agonist isoguvacine was prepared in the extracellular solution and was applied to cells by pneumatic pressure ejection. To prevent desenzitization, isoguvacine was more often ejected during 5 s at 2-min intervals. ATP, GTP, or guanosine 5′-O-(2-thiodiphosphate) (GDPβS) were administered through the patch pipette solution. We investigated the effect of competitive inhibition of GDP-GTP exchange by including GDPβS in the pipette solution (in addition to the normal amount of GTP). For GDPβS and the KIC, immediately following patch rupture, GABA current recordings were performed and the experiment was commenced after an equilibration period of 15 min.

All peptide ligands, inhibitors or allosteric modulators of the GABA_A_ receptor function were applied via gravity through a plastic tubing positioned in the vicinity of the cell body in order to maintain a sustained perfusion. In these conditions, drugs could reach the cell of interest after 30 s of perfusion. Isoguvacine was applied focally by pneumatic pressure ejection from a micropipette.

All current signals were amplified from an Axopatch 200A Amplifier (Axon Instruments, Union City, CA, USA) and filtered at 2 kHz (3 dB, four-pole, low-pass Bessel filter). Data acquisition and analysis were performed through a digidata 1200 interface using the pClamp 8 suite programs (Axon Instruments, Union City, CA, USA) and/or the Origin 4.1 analysis software (Microcal Software, Northampton, MA, USA).

The decrease of the chloride current (normalized current, I) was defined as (I-Iso+I-UII/Iso)-1 where I-Iso+I-UII is the current response in the presence of various concentrations of UII and I-Iso is the control GABA_A_R current. Concentration response curves were generated and the data were fitted by a non-linear regression analysis using Microcal Origin Software. Dose-response curves were fitted using a nonlinear square-fitting program to the equation: F(x) = Bmax/[1+(EC_50_/x)^n^], where x is the drug concentration, EC_50_ is the concentration of drug eliciting a half-maximal response and n is the Hill coefficient.

### Cell calcium imaging

For cell calcium imaging, Fura-2 AM (5 mM, Molecular Probe; Fisher, Cergy-Pontoise, France) was dissolved in 20% pluronic F-127 (w/v, DMSO) and then added to culture medium at final concentrations of 5 µM and 0.02%, with 2.5 mM probenecid (Sigma-Aldrich) respectively. Cells were incubated in the dye solution for 1 h in an humidified atmosphere (37°C, 5% CO_2_) and then rinsed in the standard extracellular solution used for patch-clamp experiments. For simultaneous measurements of intracellular calcium and chloride-evoked currents in CHO, patch-clamp electrodes were filled with an internal solution containing : KCl, 140; MgCl2, 4; Fura-2-pentoK, 0.25; HEPES, 10; ATP, 1; GTP, 0.1 (pH 7.4 adjusted with KOH). Fluorescence images were acquired with the right microscope DMLFSA (Leica) equipped with a digital CCD camera Coolsnap HQ (Photometrics, Roper scientific, Evry, France). A high-speed scanning polychromatic light source was used for alternate excitations at wavelenghts of 340 and 380 nm. The fluorescence intensities at both wavelenghts (F340 and F380) were mesured every 500 ms. Image acquisition and analysis were obtained with a MetaFluor/Metamorph Imaging System (Roper scientific). The ratio between the two images was proportional to the [Ca^2+^]_c_ in the cell under study. Before an experiment, the bath ground level of fluorescence (attributable to autofluorescence and camera noise) was determined and systematically substracted.

### Cell surface expression of receptors by ELISA

Receptor surface expression was assessed by ELISA 24 h post-transfection of CHO with cDNA encoding UT^HA^ or UT^HA^-truncated mutants (supplementary Fig. S2) or encoding UT^c-myc^ and α_2_β_3_γ_2_^HA^ GABA_A_R subunits, or U87 glioma cell line before and after *h*UII treatment, after quantification of HA, c-myc, β_3_ subunit or UT immunoreactivity. Cells were plated at 50 000 cells/well in 96-well plates coated with 0.1 mg/ml poly-L-ornithine (Sigma-Aldrich). After transfection of CHO cells with human UT^c-myc^ and α_2_β_3_γ_2_^HA^ and UT_319–389_YFP cDNA (Supplementary Table S2), cells were serum starved for 2 h before exposure to *h*UII 10^−8^ M during 30 min at 37°C. Cells were fixed with 4% PFA in PBS for 5 min at RT, washed in PBS, and then permeabilized in 0.05% triton X100 (only for permeabilized cells) and non specific binding were blocked with PBS containing 1% FBS for 30 min at RT. The first rat anti-HA monoclonal antibody (0.5 µg/ml, 3F10 clone, Roche, Meylan, France or 1 µg/ml, Santa-Cruz), mouse anti-c-myc monoclonal antibody (1.33 µg/ml, 9E10 clone, Roche), rabbit anti-UT antibody (1 µg/ml, Tebu, Santa-Cruz) or rabbit anti-β_3_ antibody (1∶200, Abcam, Paris, France), were added for 1 h30 at RT. Incubation with goat anti-rat (Thermo scientific, Fisher, Brebières, France), goat anti-mouse (Santa Cruz) or goat anti-rabbit (Tebu, Santa Cruz) conjugated alkaline phosphatase diluted at 1∶1000 in PBS/FBS was carried out for 30 min at RT. The cells were washed four times with PBS, a colorimetric alkaline phosphatase substrate was added (SuperSignal ELISA, Thermo scientific, Fisher) and the resulting color reaction was measured using a Viktor multilabel plate reader (PerkinElmer, Courtaboeuf, France). Background absorbance from samples transfected with non-tagged receptors or from cells without first antibodies were systematically measured. Results are expressed as the percentage of membrane receptor corresponding to the proportion of receptor at the plasma membrane (non permeabilized cells) to the total receptor (permeabilized cells), and normalized to the values obtained in the absence of UII. All experiments were done at least three times in triplicates.

### Receptor expression by Flow cytometry

Human astrocytes and glioma U87 grown in 75-cm2 flasks until confluence were washed in PBS, detached and spun down at 4°C. The cell pellet was washed, re-suspended in PBS containing 1% BSA and incubated with 10 µg/mL non-immune rabbit IgGs for 30 min. For total receptor detection, cells were permeabilized in PBS containing 1% BSA and 0.1% saponin for 30 min. Then, cells were incubated with antibodies directed against rabbit anti-β_3_ subunit (1∶100, Abcam) or anti-UT (1∶100, Santa Cruz), diluted in PBS containing 1% BSA and 0.1% saponin at RT (22°C) for 30 min. Non permeabilized cells were directly incubated with rabbit IgGs (10 µg/ml, 30 min), and exposed to the first antibodies for 30 min at 4°C. In negative controls, the primary antibodies were omitted. Cells were incubated with an FITC- or PerCP-conjugated goat anti-rabbit secondary antibody (Santa Cruz) diluted 1∶200 and measured in the FL1 (530 nm) or FL3 (670 nm) channel. Cells were analyzed on a FACScalibur flow cytometer (BD Biosciences) operated with the Cell QuestTM software. Samples in which the primary antibodies were omitted were used as control to normalize mean fluorescence levels. Dead cells and debris were excluded from the analysis by gating living astrocytes or glioma cells from size/structure density plots. Data were displayed on a logarithmic scale in arbitrary units corresponding to the mean fluorescence intensity. Each histogram plot was recorded from at least 10,000 gated events.

### Calcium mobilization assay

CHO cells stably transiently transfected with the human UT^HA^ or their truncated forms were plated at a density of 4×10^4^ cells/well in flat clear bottom black 96-well plates. After 24 h in culture, cells were incubated at 37°C with 40 µl of 2 µM Fluo-4 AM dye containing 20% pluronic acid for 40 min in a 5% CO_2_ atmosphere. Cells were washed twice with modified HBSS, and the effects of graded concentrations of UII on [Ca^2+^]_c_ were measured with a fluorometric imaging plate reader FlexStation II (Molecular Devices, Sunnyvale, CA) during 150 s with an excitation wavelength of 480 nm and an emission wavelength of 525 nm. After 18 s recording in basal conditions, 50 µl of graded concentration of UII (four-fold final concentration) was added to the incubation medium with a built-in eight-channel pipettor to assess activity. After subtraction of mean fluorescence background, baseline was normalized to 100%. Fluorescence peak values were determined for each concentration of UII, and potency (IC_50_) and efficacy (E_max_) were calculated with the Prism 4.0 software using a logistic equation. Results were expressed as mean ± SEM.

### Chemicals

Rat UII (*r*UII, *p*QHGTAPECFWKYCI), human UII (*h*UII, ETPDCFWKYCV), URP and [Orn^5^]URP were synthesized by the solid phase methodology on a Pioneer PerSeptive Biosystem peptide synthesizer (Applera France, Courtabœuf, France) using the standard manufacturer's procedures as previously described [32]. All peptides were purified on a 2.2×25-cm Vydac C_18_ column (Alltech, Templemars, France) (>98% pure) and characterized by MALDI-TOF MS on a Voyager DE-PRO mass spectrometer (Applera France). B27, DMEM, glutamine, HEPES, non essential amino acids, sodium pyruvate and the antibiotic-antimycotic solution were purchased from Invitrogen (Fischer, Illkirch, France). EGF was obtained from Abcys (Les Ulis, France). ATP, GTP, guanosine 5′-[βthio]triphosphate (GDPβS), the GABA_A_ receptor agonist isoguvacine, pentobarbital, poly-D-ornithine, methyl 6,7-dimethoxy-4-ethyl-β-carboline-3-carboxylate (DMCM), a phosphatase inhibitor cocktail 2 (sodium vanadate, sodium molibdate, sodium tartrate and imidazole), quercetin, staurosporine, picrotoxin, SR95531, Ham-F12, insulin, D(+)-glucose, Tri-reagent, probenicid, and bovine serum albumin (BSA) were obtained from Sigma. FBS was from Eurobio or Lonza (France). Pluronic acid and Fura-2 pentapotassium (Fura-2 AM pentoK) salt and Fura-2 acetoxymethyl ester (Fura-2 AM) were from Molecular Probes (Leiden, Netherlands). The dynamin inhibitory peptide (DIP) was obtained from Tocris Bioscience (Ellisville, MI, USA).

### Statistics

All data are presented as mean ± SEM. Statistical comparisons were assessed with One-way ANOVA followed by Mann and Whitney, Newman-Keuls or Freidman post hoc tests, as relevant, P<0.05 was taken as significance.

## Supporting Information

Figure S1 **Pharmacological and gating properties of hUII-induced regulation of GABA_A_R.** (A) Iso-evoked current in the absence or presence of pentobarbital (10^−5^ M), SR95531 (10^−5^ M) and picrotoxin (10^−4^ M, 2 s) in CHO-GABA_A_R. Right, summary of the effects of modulators on the GABAergic activity. (B) Current-Voltage (I–V) relationship of the Iso-evoked current, in the absence or presence of hUII (10^−8^ M). Data are mean ± SEM from 5 to 9 cells. *, P<0.05; **, P<0.01; ***, P<0.001 compared with the control Iso-evoked current. Ns, non significant.
(PPT)

Figure S2 **Expression of the UT C-terminus truncated mutants.** (A) Confocal microscope images of CHO expressing UT^HA^, UT_319_^HA^, UT_332_^HA^, UT_351_^HA^, UT_370_^HA^ (green). (B) Expression of the different UT^HA^ mutants expressed as receptors in whole cells (permeabilized) or only at the cell plasma membrane (non-permeabilized) using anti-HA antibody. Data are mean ± SEM from a representative experiment in triplicate. *, P<0.05; **, P<0.01; ***, P<0.001 compared to control. Mock, empty pCMV-HA vector.
(PPT)

Figure S3 **Functional expression of the UT C-terminus truncated mutants.** (A) Dose-response curves of the mean of maximum amplitude of [Ca^2+^]_c_ transients induced by *h*UII in CHO expressing UT^HA^, UT_319_^HA^, UT_332_^HA^, UT_351_^HA^, UT_370_^HA^. The results are expressed as percentages of the corresponding control values in the absence of *h*UII. (B) Corresponding table summarizing EC_50_ values and percentage of efficacy of the effect of *h*UII on each UT construction. Data are mean ± SEM from 3 independent experiments in duplicate. The Pearson coefficient *r^2^* close to 1 is used for significance.
(PPT)

Table S1 **EC_50_ and maximum inhibitory effects of** ***h*** **UII on different GABA_A_R subunit combinations.** Data are mean ± SEM from 3 to 23 independent experiments. ND, not determined; *r^2^*, Pearson coefficient.
(PPT)

Table S2 **Primer sequences and restriction enzymes used for the different UT and GABA_A_R subunit constructions.**
(PPT)

## References

[1] Takano T, Tian GF, Peng W, Lou N, Libionka W (2006). Astrocyte-mediated control of cerebral blood flow.. Nature neuroscience.

[2] Lo EH, Rosenberg GA (2009). The neurovascular unit in health and disease: introduction.. Stroke.

[3] Ohab JJ, Fleming S, Blesch A, Carmichael ST (2006). A neurovascular niche for neurogenesis after stroke.. The Journal of neuroscience : the official journal of the Society for Neuroscience.

[4] Wang DD, Bordey A (2008). The astrocyte odyssey.. Prog Neurobiol.

[5] Abbott NJ, Ronnback L, Hansson E (2006). Astrocyte-endothelial interactions at the blood-brain barrier.. Nature reviews Neuroscience.

[6] Eddleston AL (1993). Immunogenetics of autoimmune chronic active hepatitis.. Gastroenterologia Japonica.

[7] Silver J, Miller JH (2004). Regeneration beyond the glial scar.. Nature reviews Neuroscience.

[8] Seifert G, Schilling K, Steinhauser C (2006). Astrocyte dysfunction in neurological disorders: a molecular perspective.. Nature reviews Neuroscience.

[9] Sieghart W (2006). Structure, pharmacology, and function of GABAA receptor subtypes.. Adv Pharmacol.

[10] Nutt DJ, Stahl SM (2009). Searching for perfect sleep: the continuing evolution of GABAA receptor modulators as hypnotics.. J Psychopharmacol.

[11] D'Hulst C, Atack JR, Kooy RF (2009). The complexity of the GABAA receptor shapes unique pharmacological profiles.. Drug Discov Today.

[12] Von Blankenfeld G, Trotter J, Kettenmann H (1991). Expression and Developmental Regulation of a GABAA Receptor in Cultured Murine Cells of the Oligodendrocyte Lineage.. Eur J Neurosci.

[13] Fraser DD, Duffy S, Angelides KJ, Perez-Velazquez JL, Kettenmann H (1995). GABAA/benzodiazepine receptors in acutely isolated hippocampal astrocytes.. J Neurosci.

[14] Bureau M, Laschet J, Bureau-Heeren M, Hennuy B, Minet A (1995). Astroglial cells express large amounts of GABAA receptor proteins in mature brain.. J Neurochem.

[15] Tateishi N, Shimoda T, Manako J, Katsumata S, Shinagawa R (2006). Relevance of astrocytic activation to reductions of astrocytic GABAA receptors.. Brain Res.

[16] Moriwaki H, Matsumoto M, Hashikawa K, Oku N, Ishida M (1998). Iodine-123-iomazenil and iodine-123-iodoamphetamine SPECT in major cerebral artery occlusive disease.. J Nucl Med.

[17] Muller V, Saur D, Klutmann S, Weiller C, Rother J (2002). Experience with 123I-iomazenil SPECT in acute cerebral infarction.. Nucl Med Commun.

[18] Kawabata K, Tachibana H, Sugita M, Fukuchi M (1996). [Impairment of benzodiazepine receptor in Parkinson's disease evaluated by 123I-iomazenil SPECT].. Kaku Igaku.

[19] Ohyama M, Senda M, Ishiwata K, Kitamura S, Mishina M (1999). Preserved benzodiazepine receptors in Alzheimer's disease measured with C-11 flumazenil PET and I-123 iomazenil SPECT in comparison with CBF.. Ann Nucl Med.

[20] Rissman RA, Mishizen-Eberz AJ, Carter TL, Wolfe BB, De Blas AL (2003). Biochemical analysis of GABA(A) receptor subunits alpha 1, alpha 5, beta 1, beta 2 in the hippocampus of patients with Alzheimer's disease neuropathology.. Neuroscience.

[21] Verheul HB, de Leeuw FE, Scholten G, Tulleken CA, Lopes da Silva FH (1993). GABAA receptor function in the early period after transient forebrain ischaemia in the rat.. Eur J Neurosci.

[22] Luhmann HJ, Mittmann T, van Luijtelaar G, Heinemann U (1995). Impairment of intracortical GABAergic inhibition in a rat model of absence epilepsy.. Epilepsy Res.

[23] Li H, Siegel RE, Schwartz RD (1993). Rapid decline of GABAA receptor subunit mRNA expression in hippocampus following transient cerebral ischemia in the gerbil.. Hippocampus.

[24] Schwartz-Bloom RD, Sah R (2001). gamma-Aminobutyric acid(A) neurotransmission and cerebral ischemia.. J Neurochem.

[25] Labrakakis C, Patt S, Hartmann J, Kettenmann H (1998). Functional GABA(A) receptors on human glioma cells.. Eur J Neurosci.

[26] Labarrere P, Chatenet D, Leprince J, Marionneau C, Loirand G (2003). Structure-activity relationships of human urotensin II and related analogues on rat aortic ring contraction.. J Enzyme Inhib Med Chem.

[27] Sugo T, Murakami Y, Shimomura Y, Harada M, Abe M (2003). Identification of urotensin II-related peptide as the urotensin II-immunoreactive molecule in the rat brain.. Biochem Biophys Res Commun.

[28] Douglas SA, Dhanak D, Johns DG (2004). From ‘gills to pills’: urotensin-II as a regulator of mammalian cardiorenal function.. Trends Pharmacol Sci.

[29] Liu Q, Pong SS, Zeng Z, Zhang Q, Howard AD (1999). Identification of urotensin II as the endogenous ligand for the orphan G-protein-coupled receptor GPR14.. Biochem Biophys Res Commun.

[30] Elshourbagy NA, Douglas SA, Shabon U, Harrison S, Duddy G (2002). Molecular and pharmacological characterization of genes encoding urotensin-II peptides and their cognate G-protein-coupled receptors from the mouse and monkey.. Br J Pharmacol.

[31] Ziltener P, Mueller C, Haenig B, Scherz MW, Nayler O (2002). Urotensin II mediates ERK1/2 phosphorylation and proliferation in GPR14-transfected cell lines.. J Recept Signal Transduct Res.

[32] Sauzeau V, Le Mellionnec E, Bertoglio J, Scalbert E, Pacaud P (2001). Human urotensin II-induced contraction and arterial smooth muscle cell proliferation are mediated by RhoA and Rho-kinase.. Circ Res.

[33] Gong H, Wang YX, Zhu YZ, Wang WW, Wang MJ (2004). Cellular distribution of GPR14 and the positive inotropic role of urotensin II in the myocardium in adult rat.. J Appl Physiol.

[34] Johns DG, Ao Z, Naselsky D, Herold CL, Maniscalco K (2004). Urotensin-II-mediated cardiomyocyte hypertrophy: effect of receptor antagonism and role of inflammatory mediators.. Naunyn Schmiedebergs Arch Pharmacol.

[35] Shi L, Ding W, Li D, Wang Z, Jiang H (2006). Proliferation and anti-apoptotic effects of human urotensin II on human endothelial cells.. Atherosclerosis.

[36] Watanabe T, Pakala R, Katagiri T, Benedict CR (2001). Synergistic effect of urotensin II with mildly oxidized LDL on DNA synthesis in vascular smooth muscle cells.. Circulation.

[37] Guidolin D, Albertin G, Oselladore B, Sorato E, Rebuffat P (2010). The pro-angiogenic activity of urotensin-II on human vascular endothelial cells involves ERK1/2 and PI3K signaling pathways.. Regul Pept.

[38] Coulouarn Y, Lihrmann I, Jegou S, Anouar Y, Tostivint H (1998). Cloning of the cDNA encoding the urotensin II precursor in frog and human reveals intense expression of the urotensin II gene in motoneurons of the spinal cord.. Proc Natl Acad Sci U S A.

[39] Coulouarn Y, Jegou S, Tostivint H, Vaudry H, Lihrmann I (1999). Cloning, sequence analysis and tissue distribution of the mouse and rat urotensin II precursors.. FEBS Lett.

[40] Lin Y, Tsuchihashi T, Matsumura K, Fukuhara M, Ohya Y (2003). Central cardiovascular action of urotensin II in spontaneously hypertensive rats.. Hypertens Res.

[41] Castel H, Diallo M, Chatenet D, Leprince J, Desrues L (2006). Biochemical and functional characterization of high-affinity urotensin II receptors in rat cortical astrocytes.. J Neurochem.

[42] Jarry M, Diallo M, Lecointre C, Desrues L, Tokay T (2010). The vasoactive peptides urotensin II and urotensin II-related peptide regulate astrocyte activity through common and distinct mechanisms: involvement in cell proliferation.. Biochem J.

[43] Desrues L, Lefebvre T, Diallo M, Gandolfo P, Leprince J (2008). Effect of GABA A receptor activation on UT-coupled signaling pathways in rat cortical astrocytes.. Peptides.

[44] Sotelo C (2004). Cellular and genetic regulation of the development of the cerebellar system.. Prog Neurobiol.

[45] Taft JR, Vertes RP, Perry GW (2005). Distribution of GFAP+ astrocytes in adult and neonatal rat brain.. Int J Neurosci.

[46] Tretter V, Ehya N, Fuchs K, Sieghart W (1997). Stoichiometry and assembly of a recombinant GABAA receptor subtype.. J Neurosci.

[47] Blair LA, Levitan ES, Marshall J, Dionne VE, Barnard EA (1988). Single subunits of the GABAA receptor form ion channels with properties of the native receptor.. Science.

[48] Pritchett DB, Sontheimer H, Gorman CM, Kettenmann H, Seeburg PH (1988). Transient expression shows ligand gating and allosteric potentiation of GABAA receptor subunits.. Science.

[49] Sigel E, Baur R, Trube G, Mohler H, Malherbe P (1990). The effect of subunit composition of rat brain GABAA receptors on channel function.. Neuron.

[50] Krishek BJ, Xie X, Blackstone C, Huganir RL, Moss SJ (1994). Regulation of GABAA receptor function by protein kinase C phosphorylation.. Neuron.

[51] Angelotti TP, Macdonald RL (1993). Assembly of GABAA receptor subunits: alpha 1 beta 1 and alpha 1 beta 1 gamma 2S subunits produce unique ion channels with dissimilar single-channel properties.. J Neurosci.

[52] Diallo M, Jarry M, Desrues L, Castel H, Chatenet D (2008). [Orn5]URP acts as a pure antagonist of urotensinergic receptors in rat cortical astrocytes.. Peptides.

[53] Chatenet D, Dubessy C, Leprince J, Boularan C, Carlier L (2004). Structure-activity relationships and structural conformation of a novel urotensin II-related peptide.. Peptides.

[54] Clozel M, Binkert C, Birker-Robaczewska M, Boukhadra C, Ding SS (2004). Pharmacology of the urotensin-II receptor antagonist palosuran (ACT-058362; 1-[2-(4-benzyl-4-hydroxy-piperidin-1-yl)-ethyl]-3-(2-methyl-quinolin-4-yl) -urea sulfate salt): first demonstration of a pathophysiological role of the urotensin System.. J Pharmacol Exp Ther.

[55] Behm DJ, McAtee JJ, Dodson JW, Neeb MJ, Fries HE (2008). Palosuran inhibits binding to primate UT receptors in cell membranes but demonstrates differential activity in intact cells and vascular tissues.. Br J Pharmacol.

[56] Puia G, Vicini S, Seeburg PH, Costa E (1991). Influence of recombinant gamma-aminobutyric acid-A receptor subunit composition on the action of allosteric modulators of gamma-aminobutyric acid-gated Cl- currents.. Mol Pharmacol.

[57] Wafford KA, Whiting PJ, Kemp JA (1993). Differences in affinity and efficacy of benzodiazepine receptor ligands at recombinant gamma-aminobutyric acidA receptor subtypes.. Mol Pharmacol.

[58] Bianchi MT, Haas KF, Macdonald RL (2001). Structural determinants of fast desensitization and desensitization-deactivation coupling in GABAa receptors.. The Journal of neuroscience : the official journal of the Society for Neuroscience.

[59] Boileau AJ, Baur R, Sharkey LM, Sigel E, Czajkowski C (2002). The relative amount of cRNA coding for gamma2 subunits affects stimulation by benzodiazepines in GABA(A) receptors expressed in Xenopus oocytes.. Neuropharmacology.

[60] Yoon KW (1994). Voltage-dependent modulation of GABAA receptor channel desensitization in rat hippocampal neurons.. Journal of neurophysiology.

[61] Dominguez-Perrot C, Feltz P, Poulter MO (1996). Recombinant GABAA receptor desensitization: the role of the gamma 2 subunit and its physiological significance.. The Journal of physiology.

[62] Moss SJ, Smart TG (1996). Modulation of amino acid-gated ion channels by protein phosphorylation.. Int Rev Neurobiol.

[63] Brandon N, Jovanovic J, Moss S (2002). Multiple roles of protein kinases in the modulation of gamma-aminobutyric acid(A) receptor function and cell surface expression.. Pharmacol Ther.

[64] Kittler JT, Moss SJ (2003). Modulation of GABAA receptor activity by phosphorylation and receptor trafficking: implications for the efficacy of synaptic inhibition.. Curr Opin Neurobiol.

[65] Gartlon J, Parker F, Harrison DC, Douglas SA, Ashmeade TE (2001). Central effects of urotensin-II following ICV administration in rats.. Psychopharmacology (Berl).

[66] Jegou S, Cartier D, Dubessy C, Gonzalez BJ, Chatenet D (2006). Localization of the urotensin II receptor in the rat central nervous system.. J Comp Neurol.

[67] Clark SD, Nothacker HP, Wang Z, Saito Y, Leslie FM (2001). The urotensin II receptor is expressed in the cholinergic mesopontine tegmentum of the rat.. Brain Res.

[68] Pirker S, Schwarzer C, Wieselthaler A, Sieghart W, Sperk G (2000). GABA(A) receptors: immunocytochemical distribution of 13 subunits in the adult rat brain.. Neuroscience.

[69] Fritschy JM, Panzanelli P (2006). Molecular and synaptic organization of GABAA receptors in the cerebellum: Effects of targeted subunit gene deletions.. Cerebellum.

[70] Laurie DJ, Wisden W, Seeburg PH (1992). The distribution of thirteen GABAA receptor subunit mRNAs in the rat brain. III. Embryonic and postnatal development.. J Neurosci.

[71] Bovolin P, Santi MR, Puia G, Costa E, Grayson D (1992). Expression patterns of gamma-aminobutyric acid type A receptor subunit mRNAs in primary cultures of granule neurons and astrocytes from neonatal rat cerebella.. Proc Natl Acad Sci USA.

[72] McKhann GM, D'Ambrosio R, Janigro D (1997). Heterogeneity of astrocyte resting membrane potentials and intercellular coupling revealed by whole-cell and gramicidin-perforated patch recordings from cultured neocortical and hippocampal slice astrocytes.. J Neurosci.

[73] Haydar TF, Wang F, Schwartz ML, Rakic P (2000). Differential modulation of proliferation in the neocortical ventricular and subventricular zones.. J Neurosci.

[74] LoTurco JJ, Owens DF, Heath MJ, Davis MB, Kriegstein AR (1995). GABA and glutamate depolarize cortical progenitor cells and inhibit DNA synthesis.. Neuron.

[75] Nilsson M, Eriksson PS, Ronnback L, Hansson E (1993). GABA induces Ca2+ transients in astrocytes.. Neuroscience.

[76] Ames RS, Sarau HM, Chambers JK, Willette RN, Aiyar NV (1999). Human urotensin-II is a potent vasoconstrictor and agonist for the orphan receptor GPR14.. Nature.

[77] Mori M, Sugo T, Abe M, Shimomura Y, Kurihara M (1999). Urotensin II is the endogenous ligand of a G-protein-coupled orphan receptor, SENR (GPR14).. Biochem Biophys Res Commun.

[78] Nothacker HP, Wang Z, McNeill AM, Saito Y, Merten S (1999). Identification of the natural ligand of an orphan G-protein-coupled receptor involved in the regulation of vasoconstriction.. Nat Cell Biol.

[79] Qi J, Du J, Tang X, Li J, Wei B (2004). The upregulation of endothelial nitric oxide synthase and urotensin-II is associated with pulmonary hypertension and vascular diseases in rats produced by aortocaval shunting.. Heart Vessels.

[80] Green SA, Spasoff AP, Coleman RA, Johnson M, Liggett SB (1996). Sustained activation of a G protein-coupled receptor via “anchored" agonist binding. Molecular localization of the salmeterol exosite within the 2-adrenergic receptor.. J Biol Chem.

[81] Jakubik J, Tucek S, El-Fakahany EE (2002). Allosteric modulation by persistent binding of xanomeline of the interaction of competitive ligands with the M1 muscarinic acetylcholine receptor.. J Pharmacol Exp Ther.

[82] Ono T, Kawaguchi Y, Kudo M, Kushikata T, Hashiba E (2008). Urotensin II evokes neurotransmitter release from rat cerebrocortical slices.. Neurosci Lett.

[83] Pryor PR, Mullock BM, Bright NA, Gray SR, Luzio JP (2000). The role of intraorganellar Ca(2+) in late endosome-lysosome heterotypic fusion and in the reformation of lysosomes from hybrid organelles.. J Cell Biol.

[84] Lin FT, Krueger KM, Kendall HE, Daaka Y, Fredericks ZL (1997). Clathrin-mediated endocytosis of the beta-adrenergic receptor is regulated by phosphorylation/dephosphorylation of beta-arrestin1.. J Biol Chem.

[85] Herring D, Huang R, Singh M, Robinson LC, Dillon GH (2003). Constitutive GABAA receptor endocytosis is dynamin-mediated and dependent on a dileucine AP2 adaptin-binding motif within the beta 2 subunit of the receptor.. J Biol Chem.

[86] Naga Prasad SV, Jayatilleke A, Madamanchi A, Rockman HA (2005). Protein kinase activity of phosphoinositide 3-kinase regulates beta-adrenergic receptor endocytosis.. Nat Cell Biol.

[87] Barnes EM (2001). Assembly and intracellular trafficking of GABAA receptors.. Int Rev Neurobiol.

[88] Proulx CD, Simaan M, Escher E, Laporte SA, Guillemette G (2005). Involvement of a cytoplasmic-tail serine cluster in urotensin II receptor internalization.. Biochem J.

[89] Cao W, Luttrell LM, Medvedev AV, Pierce KL, Daniel KW (2000). Direct binding of activated c-Src to the beta 3-adrenergic receptor is required for MAP kinase activation.. J Biol Chem.

[90] Gandolfo P, Patte C, Leprince J, Thoumas JL, Vaudry H (1997). The stimulatory effect of the octadecaneuropeptide (ODN) on cytosolic Ca2+ in rat astrocytes is not mediated through classical benzodiazepine receptors.. Eur J Pharmacol.

[91] Lefebvre T, Gonzalez BJ, Vaudry D, Desrues L, Falluel-Morel A (2009). Paradoxical effect of ethanol on potassium channel currents and cell survival in cerebellar granule neurons.. J Neurochem.

