# Supplementary material for: Down-Regulation of GABAA Receptor via Promiscuity with the Vasoactive Peptide Urotensin II Receptor. Potential Involvement in Astrocyte Plasticity
Source: PLoS One. 2012 May 1;7(5):e36319. doi: 10.1371/journal.pone.0036319 (PMC3341351; doi:10.1371/journal.pone.0036319)
Supplement: Figure S1 — Pharmacological and gating properties of hUII-induced regulation of GABAAR. (A) Iso-evoked current in the absence or presence of pentobarbital (10−5 M), SR95531 (10−5 M) and picrotoxin (10−4 M, 2 s) in CHO-GABAAR. Right, summary of the effects of modulators on the GABAergic activity. (B) Current-Voltage (I–V) relationship of the Iso-evoked current, in the absence or presence of hUII (10−8 M). Data are mean ± SEM from 5 to 9 cells. *, P<0.05; **, P<0.01; ***, P<0.001 compared with the control Iso-evoked current. Ns, non significant. (PPT) [file pone.0036319.s001.ppt]

## Slide 1
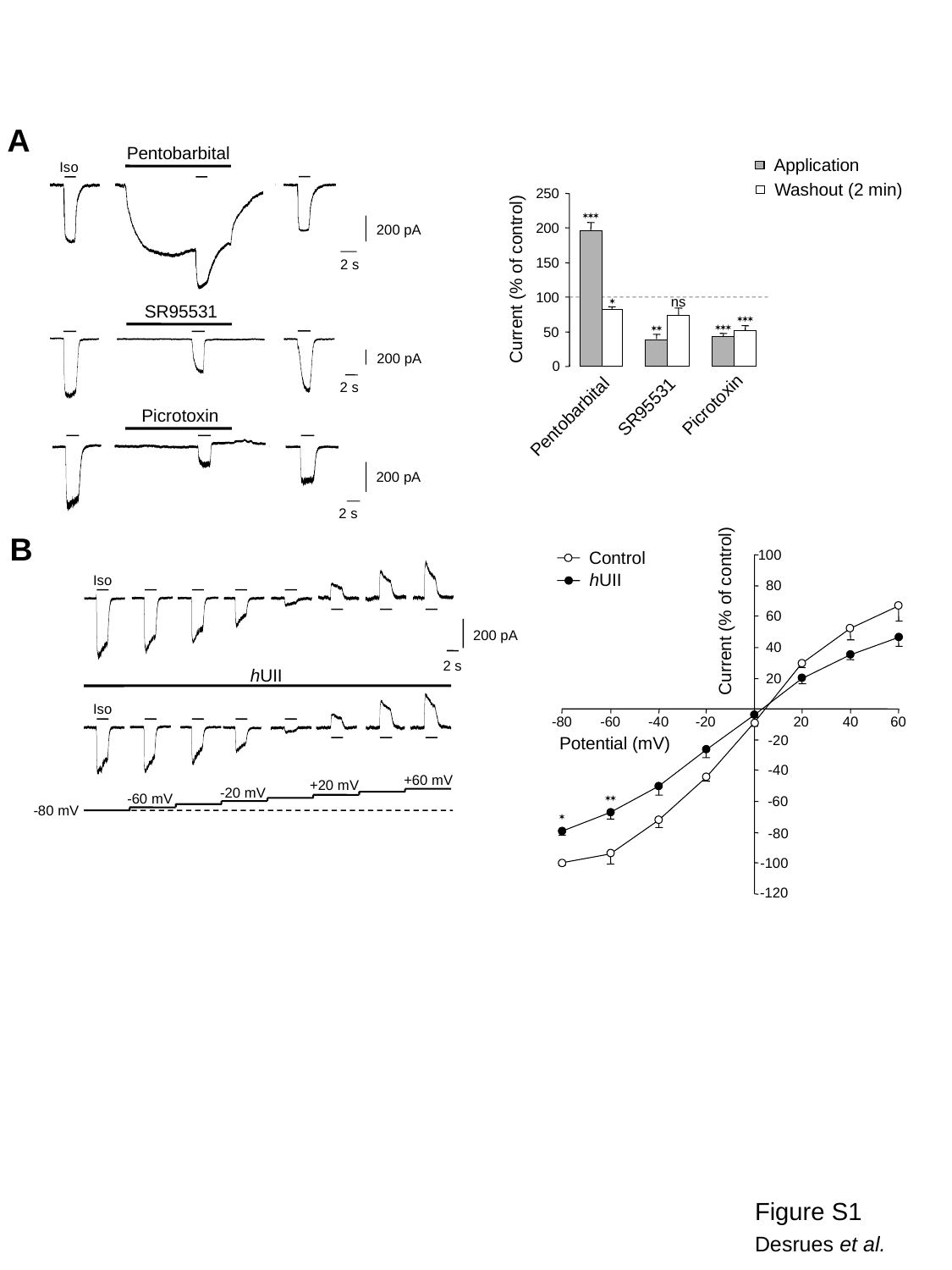

A
Pentobarbital
Iso
Application
Washout (2 min)
250

200 pA
200
2 s
150
Current (% of control)
100
ns
SR95531




50
200 pA
0
2 s
Picrotoxin
SR95531
Picrotoxin
Pentobarbital
200 pA
2 s
Control
100
hUII
80
Current (% of control)
60
40
20
-80
-60
-40
-20
20
40
60
Potential (mV)
-20
-40

-60

-80
-100
-120
B
Iso
200 pA
2 s
hUII
Iso
+60 mV
+20 mV
-20 mV
-60 mV
-80 mV
Figure S1
Desrues et al.
