# Supplementary material for: Down-Regulation of GABAA Receptor via Promiscuity with the Vasoactive Peptide Urotensin II Receptor. Potential Involvement in Astrocyte Plasticity
Source: PLoS One. 2012 May 1;7(5):e36319. doi: 10.1371/journal.pone.0036319 (PMC3341351; doi:10.1371/journal.pone.0036319)
Supplement: Figure S2 — Expression of the UT C-terminus truncated mutants. (A) Confocal microscope images of CHO expressing UTHA, UT319 HA, UT332 HA, UT351 HA, UT370 HA (green). (B) Expression of the different UTHA mutants expressed as receptors in whole cells (permeabilized) or only at the cell plasma membrane (non-permeabilized) using anti-HA antibody. Data are mean ± SEM from a representative experiment in triplicate. *, P<0.05; **, P<0.01; ***, P<0.001 compared to control. Mock, empty pCMV-HA vector. (PPT) [file pone.0036319.s002.ppt]

## Slide 1
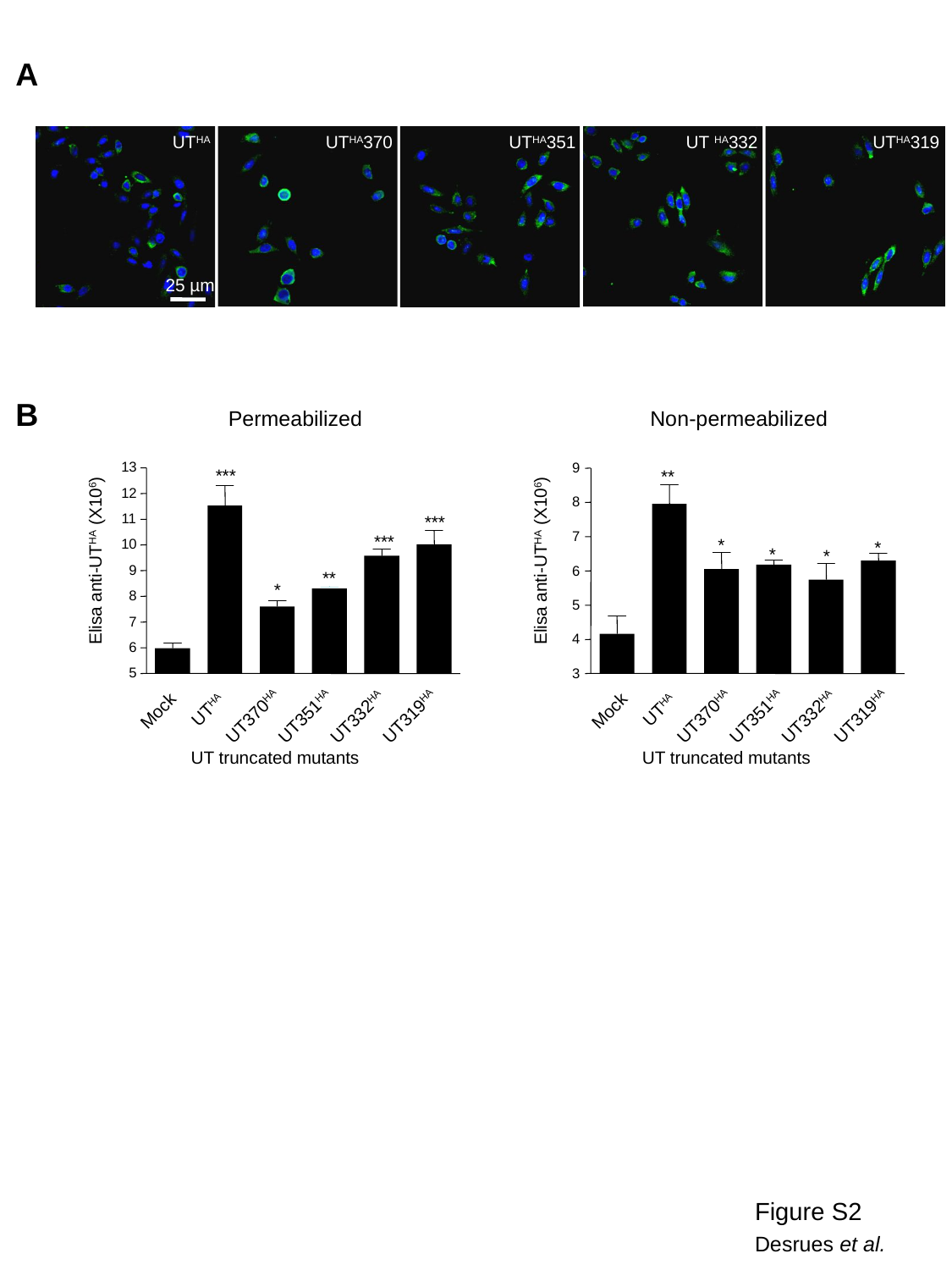

A
UTHA
UTHA370
UTHA351
UT HA332
UTHA319
25 µm
B
Permeabilized
Non-permeabilized
***
13
9
**
12
8
***
11
***
*
7
*
10
*
*
Elisa anti-UTHA (X106)
Elisa anti-UTHA (X106)
**
9
6
*
8
5
7
4
6
5
3
UTHA
Mock
UT370HA
UT351HA
UT319HA
UT332HA
UT truncated mutants
UTHA
Mock
UT370HA
UT351HA
UT319HA
UT332HA
UT truncated mutants
Figure S2
Desrues et al.
