# Supplementary material for: Down-Regulation of GABAA Receptor via Promiscuity with the Vasoactive Peptide Urotensin II Receptor. Potential Involvement in Astrocyte Plasticity
Source: PLoS One. 2012 May 1;7(5):e36319. doi: 10.1371/journal.pone.0036319 (PMC3341351; doi:10.1371/journal.pone.0036319)
Supplement: Figure S3 — Functional expression of the UT C-terminus truncated mutants. (A) Dose-response curves of the mean of maximum amplitude of [Ca2+]c transients induced by hUII in CHO expressing UTHA, UT319 HA, UT332 HA, UT351 HA, UT370 HA. The results are expressed as percentages of the corresponding control values in the absence of hUII. (B) Corresponding table summarizing EC50 values and percentage of efficacy of the effect of hUII on each UT construction. Data are mean ± SEM from 3 independent experiments in duplicate. The Pearson coefficient r2 close to 1 is used for significance. (PPT) [file pone.0036319.s003.ppt]

## Slide 1
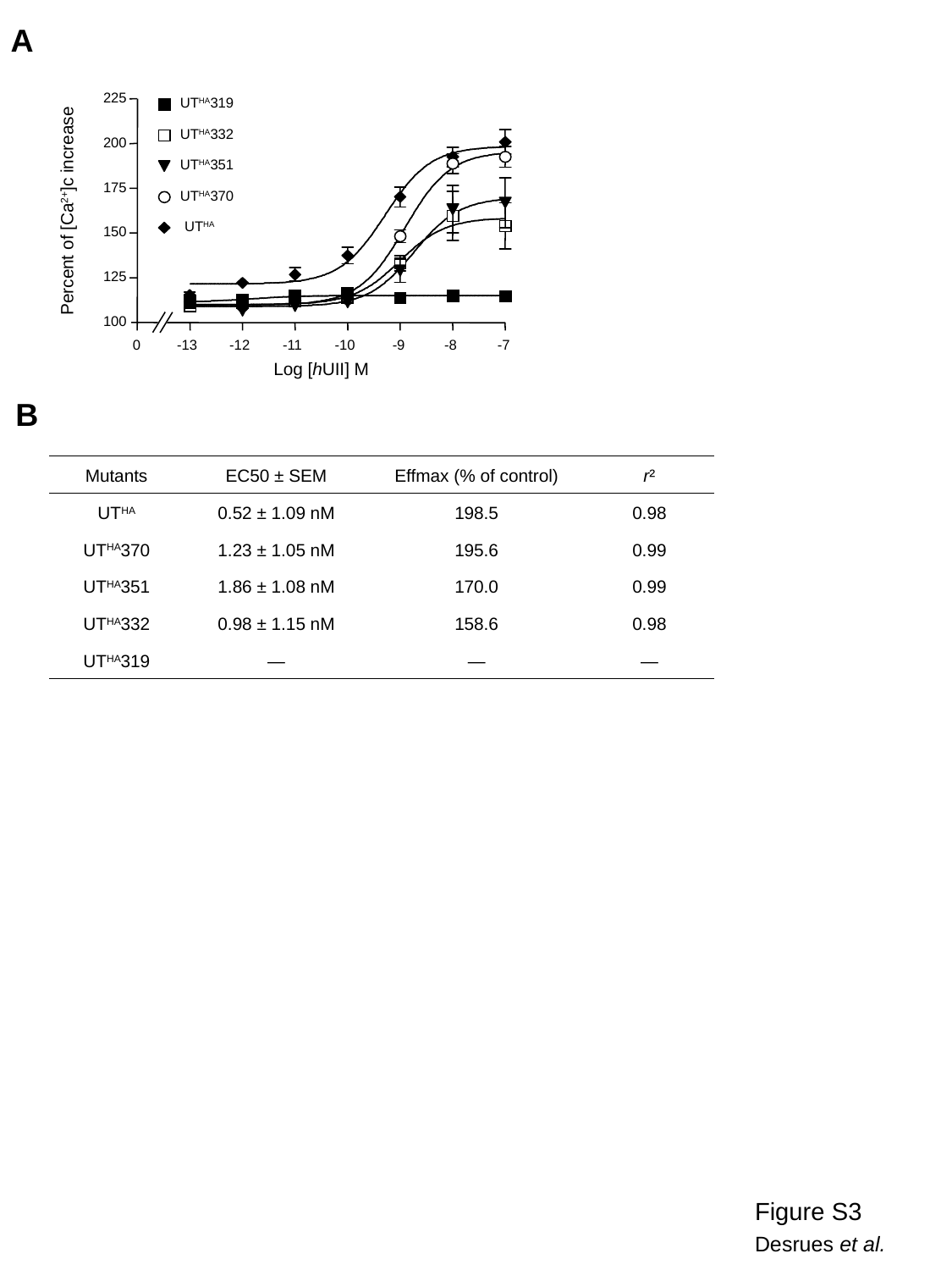

A
225
UTHA319
UTHA332
200
UTHA351
175
UTHA370
Percent of [Ca2+]c increase
UTHA
150
125
100
0
-13
-12
-11
-10
-9
-8
-7
Log [hUII] M
B
| Mutants | EC50 ± SEM | Effmax (% of control) | r² |
| --- | --- | --- | --- |
| UTHA | 0.52 ± 1.09 nM | 198.5 | 0.98 |
| UTHA370 | 1.23 ± 1.05 nM | 195.6 | 0.99 |
| UTHA351 | 1.86 ± 1.08 nM | 170.0 | 0.99 |
| UTHA332 | 0.98 ± 1.15 nM | 158.6 | 0.98 |
| UTHA319 | — | — | — |
Figure S3
Desrues et al.
