# Supplementary material for: Down-Regulation of GABAA Receptor via Promiscuity with the Vasoactive Peptide Urotensin II Receptor. Potential Involvement in Astrocyte Plasticity
Source: PLoS One. 2012 May 1;7(5):e36319. doi: 10.1371/journal.pone.0036319 (PMC3341351; doi:10.1371/journal.pone.0036319)
Supplement: Table S1 — EC50 and maximum inhibitory effects of h UII on different GABAAR subunit combinations. Data are mean ± SEM from 3 to 23 independent experiments. ND, not determined; r2, Pearson coefficient. (PPT) [file pone.0036319.s004.ppt]

## Slide 1
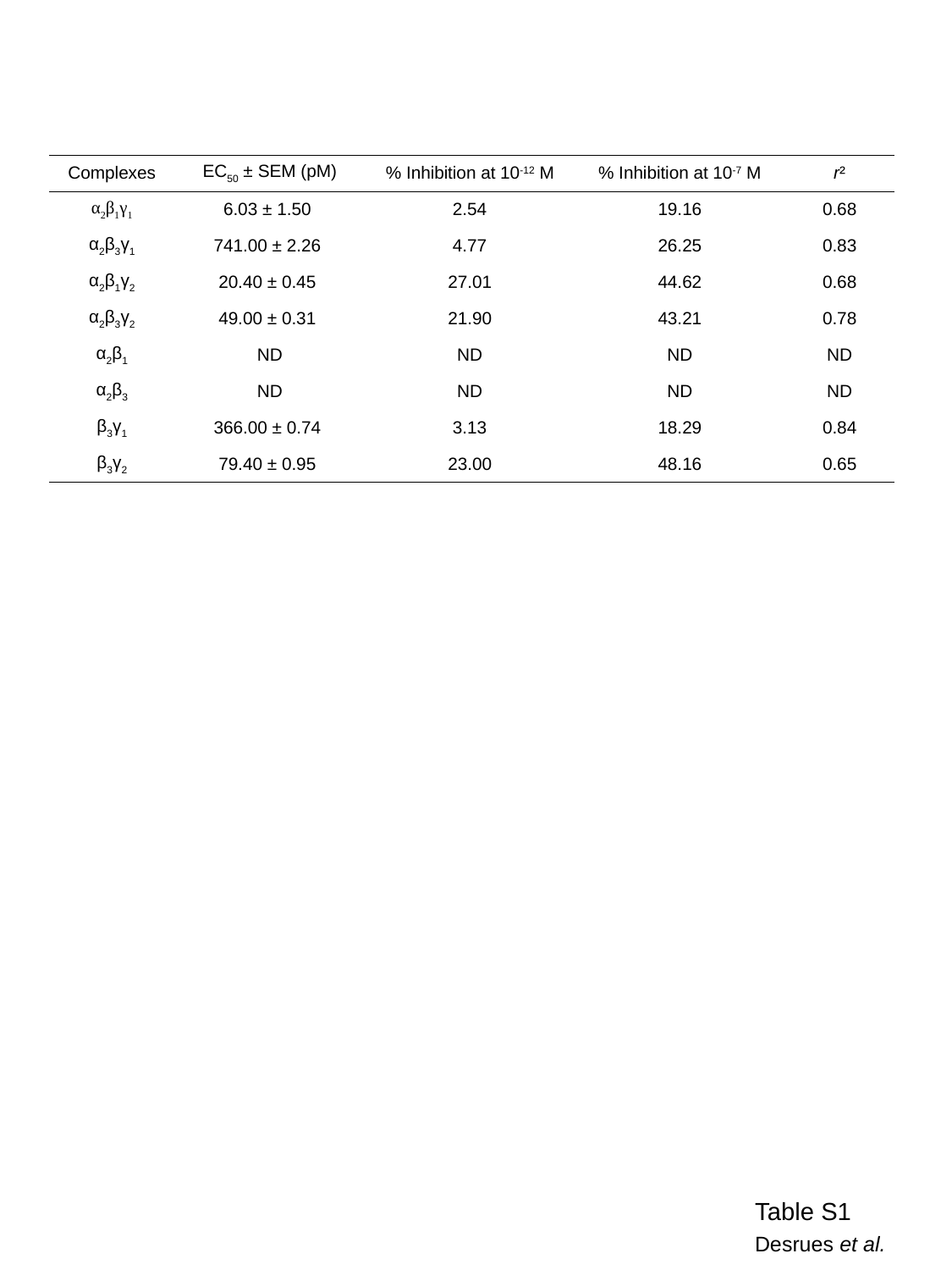

| Complexes | EC50 ± SEM (pM) | % Inhibition at 10-12 M | % Inhibition at 10-7 M | r² |
| --- | --- | --- | --- | --- |
| α2β1γ1 | 6.03 ± 1.50 | 2.54 | 19.16 | 0.68 |
| α2β3γ1 | 741.00 ± 2.26 | 4.77 | 26.25 | 0.83 |
| α2β1γ2 | 20.40 ± 0.45 | 27.01 | 44.62 | 0.68 |
| α2β3γ2 | 49.00 ± 0.31 | 21.90 | 43.21 | 0.78 |
| α2β1 | ND | ND | ND | ND |
| α2β3 | ND | ND | ND | ND |
| β3γ1 | 366.00 ± 0.74 | 3.13 | 18.29 | 0.84 |
| β3γ2 | 79.40 ± 0.95 | 23.00 | 48.16 | 0.65 |
Table S1
Desrues et al.
