# Supplementary figures and images for: Down-Regulation of GABAA Receptor via Promiscuity with the Vasoactive Peptide Urotensin II Receptor. Potential Involvement in Astrocyte Plasticity
Source: PLoS One. 2012 May 1;7(5):e36319. doi: 10.1371/journal.pone.0036319 (PMC3341351; doi:10.1371/journal.pone.0036319)

## Slide 1
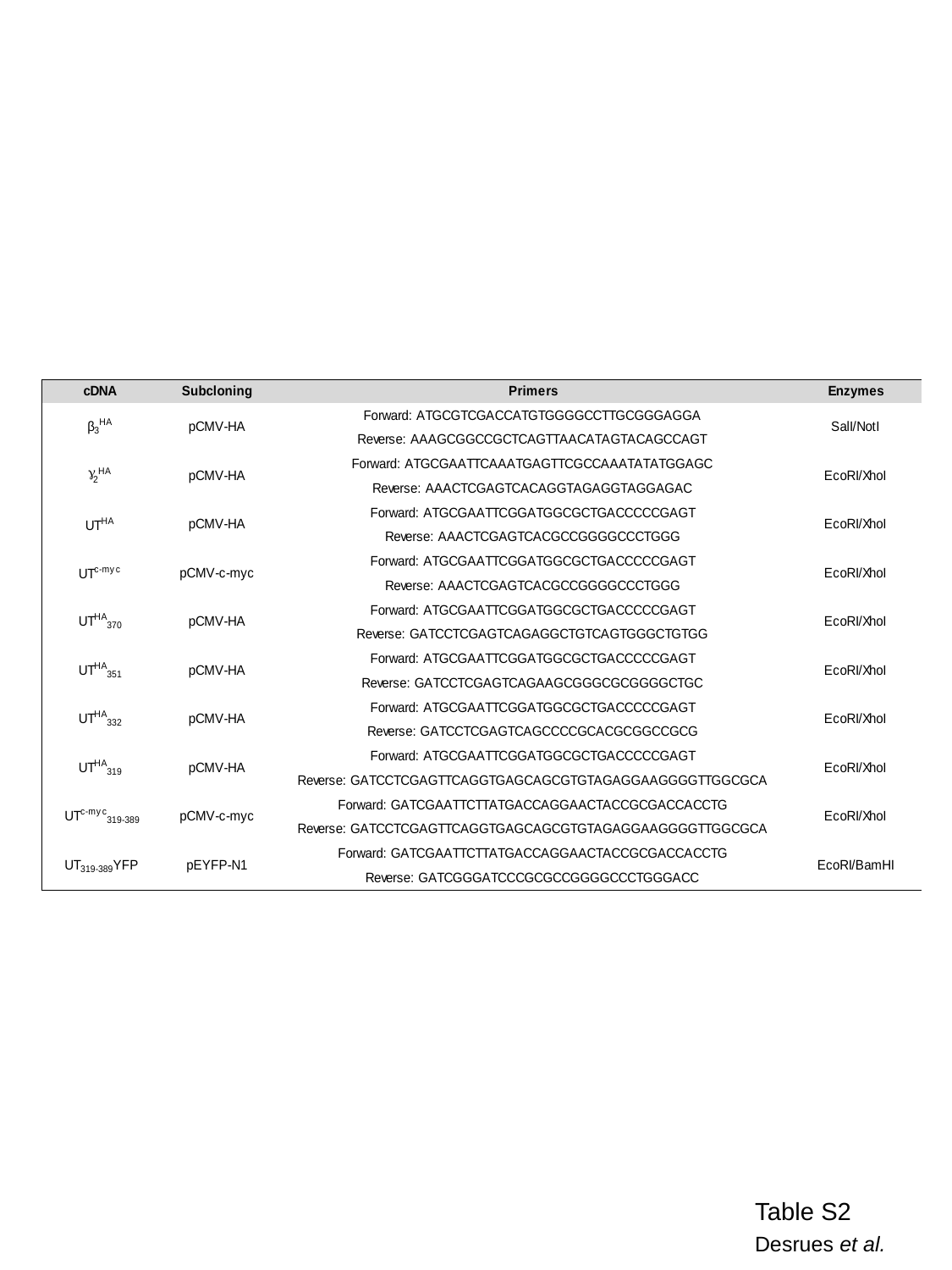

Table S2
Desrues et al.

Supplement: Table S2 — Primer sequences and restriction enzymes used for the different UT and GABAAR subunit constructions. (PPT) [file pone.0036319.s005.ppt]
